# Complete genome sequence and analysis of *Alcaligenes faecalis* strain Mc250, a new potential plant bioinoculant

**DOI:** 10.1371/journal.pone.0241546

**Published:** 2020-11-05

**Authors:** Érica Barbosa Felestrino, Angélica Bianchini Sanchez, Washington Luiz Caneschi, Camila Gracyelle de Carvalho Lemes, Renata de Almeida Barbosa Assis, Isabella Ferreira Cordeiro, Natasha Peixoto Fonseca, Morghana Marina Villa, Izadora Tabuso Vieira, Luciana Hiromi Yoshino Kamino, Flávio Fonseca do Carmo, Aline Maria da Silva, Andrew Maltez Thomas, José Salvatore Leister Patané, Fernanda Carla Ferreira, Leandro Grassi de Freitas, Alessandro de Mello Varani, Jesus Aparecido Ferro, Robson Soares Silva, Nalvo Franco Almeida, Camila Carrião Machado Garcia, João Carlos Setubal, Leandro Marcio Moreira

**Affiliations:** 1 Núcleo de Pesquisas em Ciências Biológicas (NUPEB), Universidade Federal de Ouro Preto, Ouro Preto, MG, Brazil; 2 Departamento de Ciências Biológicas (DECBI), Instituto de Ciências Exatas e Biológicas (ICEB), Universidade Federal de Ouro Preto (UFOP), Ouro Preto, MG, Brazil; 3 Instituto Prístino, Belo Horizonte, MG, Brazil; 4 Departamento de Bioquímica (DBQ), Instituto de Química (IQ), Universidade de São Paulo (USP), São Paulo, SP, Brazil; 5 Instituto de Biotecnologia Aplicada a Agropecuária (BIOAGRO), Universidade Federal de Viçosa (UFV), Viçosa, MG, Brazil; 6 Departamento de Tecnologia, Faculdade de Ciências Agrárias e Veterinárias de Jaboticabal (FCAV), Universidade Estadual Paulista (UNESP), São Paulo, SP, Brazil; 7 Faculdade de Computação (FACOM), Universidade Federal de Mato Grosso do Sul, Campo Grande, MS, Brazil; Instituto Butantan, BRAZIL

## Abstract

Here we present and analyze the complete genome of *Alcaligenes faecalis* strain Mc250 (Mc250), a bacterium isolated from the roots of *Mimosa calodendron*, an endemic plant growing in ferruginous rupestrian grasslands in Minas Gerais State, Brazil. The genome has 4,159,911 bp and 3,719 predicted protein-coding genes, in a single chromosome. Comparison of the Mc250 genome with 36 other *Alcaligenes faecalis* genomes revealed that there is considerable gene content variation among these strains, with the core genome representing only 39% of the protein-coding gene repertoire of Mc250. Mc250 encodes a complete denitrification pathway, a network of pathways associated with phenolic compounds degradation, and genes associated with HCN and siderophores synthesis; we also found a repertoire of genes associated with metal internalization and metabolism, sulfate/sulfonate and cysteine metabolism, oxidative stress and DNA repair. These findings reveal the genomic basis for the adaptation of this bacterium to the harsh environmental conditions from where it was isolated. Gene clusters associated with ectoine, terpene, resorcinol, and emulsan biosynthesis that can confer some competitive advantage were also found. Experimental results showed that Mc250 was able to reduce (~60%) the virulence phenotype of the plant pathogen *Xanthomonas citri* subsp. *citri* when co-inoculated in *Citrus sinensis*, and was able to eradicate 98% of juveniles and stabilize the hatching rate of eggs to 4% in two species of agricultural nematodes. These results reveal biotechnological potential for the Mc250 strain and warrant its further investigation as a biocontrol and plant growth-promoting bacterium.

## Introduction

The *Alcaligenes faecalis* (*Af*) species comprises of rod shaped, Gram-negative, aerobic and polyvitric strains that have optimal growth at temperatures ranging from 20 to 37°C [1]. This bacteria is widely found in water and soil samples and have been shown to be causal agents of opportunistic pathologies in humans and animals [2, 3]. Biochemical and molecular studies have demonstrated that some strains of *Af* have biosurfactant production potential [4], ability to act as denitrifying organisms [5, 6], high arsenic oxidizing capacity [7–9], and ability to act as biocontrol of nematodes and insects due to their high killing potential against some species of these agricultural pests [10–12]. In summary, *Af* strains have been shown to be valuable as important biofertilizer, bioremediation, and biocontrol agents.

Several *Af* genomes have been sequenced [11, 13–18]. However, the first comparative analysis of *Af* genomes was published only recently, focusing on the analysis of systems related to antibiotic, metal, and pollutant resistance [13].

A previous work by our group described *Af* strain Mc250 (Mc250) isolated from *Mimosa calodendron* (Fabaceae) roots as part of a prospection study of bacteria associated with plants endemic to ferruginous rupestrian grasslands of the Brazilian Iron Quadrangle [19]. This strain was shown to have high potential as a plant growth promoting bacterium (PGPB), acting mainly as a rhizoremediator of arsenic-contaminated soil [19]. These initial results along with the biotechnological potential reported for strains of this species prompted us to sequence the Mc250 genome and perform a detailed comparison with other published genomes of this species. We identified several metabolic pathways understudied in *Af* such as those associated with degradation of phenolic compounds, plant hormone synthesis pathways, and pathways related to biomolecules that aid in plant development and those that have the potential to inhibit different plant pathogens and agricultural pests. The latter feature led us to experimentally investigate the inhibitory effects that Mc250 might have against the plant pathogen *Xanthomonas citri* subsp. *citri* and two nematodes species that are also plant pathogens, with positive results.

## Materials and methods

### Ethics statement

The field research was approved by the Ministério do Meio Ambiente—MMA; Instituto Chico Mendes de Conservação da Biodiversidade—ICMBio; Sistema de Autorização e Informação em Biodiversidade–SISBIO, field permit number 54015.

### Bacterial DNA extraction, sequencing and genome assembly

The strain *Alcaligenes faecalis* Mc250 (Mc250) was isolated from *Mimosa calodendron*, an endemic plant of the ferruginous rupestrian grasslands from the Iron Quadrangle [19]. Mc250 was grown in 50 mL of Luria Bertani medium (10 g/L peptone, 10 g/L NaCl, 5 g/L yeast extract, pH 7.0) for 2 days at 28°C under agitation of 220 rpm, and DNA was extracted using the Wizard Genomic DNA purification™ kit (Promega) according to product specifications. DNA integrity was examined with DNA 7500 chip using 2100 Bioanalyzer, revealing an enrichment of fragments higher than 10 kbp. Sequencing library was prepared with Illumina Nextera DNA library preparation kit (Illumina, Inc., USA) with a total DNA input of 40 ng. After quantification with the KAPA Library Quantification Kit, the library was subjected to one run using the MiSeq Reagent kit v2 (500-cycle format, paired-end (PE) reads). On average, Illumina PE read1 and read2 presented, respectively, >80% and >75% of bases with quality score at least 30 (Q30). Raw reads were trimmed with Trimmomatic v0.35 [20] and assembled with SPAdes v3.12.0 [21]. In addition, the MaSuRCA assembler v3.2.6 [22] was also used. The use of and comparison of different genome assembly algorithm results generally leads to the resolution of the rRNA operons copies and other smaller repeats, permitting the manual extension and junctions of contigs. Therefore, the final genome sequence was generated by the comparisons of both SPAdes and MaSuRCA assembly results by the use of the cross_match software (http://www.phrap.org), platanus scaffold and gap_close v1.2.4 [23] and special scripts. The trimmed reads were mapped back to the final genome sequence with bowtie2 v2.3.4.1 [24] and the estimated paired-end reads distance were inspected in order to verify misassembled regions and low covered regions. A total of 99.81% of the paired-reads were aligned concordantly, thus supporting a high confidence, complete and circular genome, with an average coverage of ~250x.

### Genome availability

The sequence of the Mc250 genome is available at GenBank under accession number NZ_CP031012.1, Bioproject PRJNA481026 and Biosample SAMN09655358.

### Phylogenomic analysis

A database containing 38 genomes was built (http://jau.facom.ufms.br/alcaligenes6/), 36 of which are genomes of *Alcaligenes faecalis* (including Mc250), one is the genome of *Alcaligenes aquatilis*, and one is the genome of *Paenalcaligenes hominis* strain 15S00501 (GCA_002005365.1), used as an outgroup, but also belonging to the Alcaligenaceae family (Table 1). All genomes were annotated with Prokka [25].

**Table 1:** List of 38 *Alcaligenes* genomes (clade ID 21179) used in the phylogenomic analyses.

Organism Name	Strain	Bio Sample	Bio Project	Assembly	Size (Mb)	GC%	RefSeq or WGS	Scaffolds	CDS	Release Date	Level
*Alcaligenes faecalis*	ZD02	SAMN03379009	PRJNA276624	GCA_000967305.2	4.24868	56.8	chr: NZ_CP013119.1	2	3766	16/11/2015	Complete
							pl: pZD02: NZ_CP013143.1				
*Alcaligenes faecalis*	DSM 30030	SAMN07701806	PRJNA412153	GCA_002443155.1	4.07526	56.6	chr: NZ_CP023667.1	1	3656	03/10/2017	Complete
*Alcaligenes faecalis*	MUB14	SAMN13899430	PRJNA602591	GCA_010092625.1	4.45594	56.7	chr: NZ_CP048039.1	3	3971	01/02/2020	Complete
							pl: pMUB-AF14-1:NZ_CP048040.1				
							pl: pMUB-AF14-2:NZ_CP048041.1				
*Alcaligenes faecalis*	JQ135	SAMN07173706	PRJNA388257	GCA_002242175.1	4.07835	55.9	chr: NZ_CP021641.1	1	3596	06/08/2017	Complete
*Alcaligenes faecalis*	FDAARGOS_491	SAMN10163167	PRJNA231221	GCA_003813085.1	4.0729	56.6	chr: NZ_CP033861.1	1	3650	20/11/2018	Complete
*Alcaligenes faecalis*	P156	SAMN04505616	PRJNA312705	GCA_001641975.2	4.04751	56.7	chr: NZ_CP021079.1	1	3597	30/04/2017	Complete
*Alcaligenes faecalis*	AN70	SAMN10964208	PRJNA523123	GCA_004319585.1	3.92272	57.3	chr: NZ_CP036294.1	1	3504	27/02/2019	Complete
*Alcaligenes faecalis*	J481	SAMN10101723	PRJNA492379	GCA_003716855.1	3.8668	55.7	chr: NZ_CP032521.1	1	3392	06/11/2010	Complete
*Alcaligenes faecalis*	Mc250	SAMN09655358	PRJNA481026	GCA_009497775.1	4.15991	56.7	chr: NZ_CP031012.1	1	3719	03/11/2019	Chr
*Alcaligenes faecalis*	AU14	SAMN09841847	PRJNA486231	GCA_005311025.1	4.20424	56.4	chr: NZ_CP031747.1	1	3748	13/05/2019	Chr
*Alcaligenes faecalis*	BDB4	SAMN07187750	PRJNA388846	GCA_002205415.1	4.23698	56.0	chr: NZ_CP021883.1	2	3081	21/06/2017	Chr
							pl: pZD02:NZ_CP021884.1				
*Alcaligenes faecalis subsp*. *faecalis NBRC 13111*	NBRC 13111	SAMD00058712	PRJDB5133	GCA_001748345.1	4.04291	56.7	BDHG01	27	3644	01/09/2016	Contig
*Alcaligenes faecalis subsp*. *faecalis NBRC 13111*	NBRC 13111	SAMD00000359	PRJDB275	GCA_000739855.1	4.03369	56.7	BBJQ01	29	3646	01/08/2014	Contig
*Alcaligenes faecalis*	ATCC 8750	SAMEA2247602	PRJEB4659	GCA_001298815.1	4.03996	56.7	CYTB01	24	3646	18/09/2015	Scaffold
*Alcaligenes faecalis*	YBY	SAMN09080444	PRJNA464269	GCA_003122065.1	4.32793	56.6	QEXO01	11	3868	11/05/2018	Contig
*Alcaligenes faecalis subsp*. *phenolicus*	MB207	SAMN06212345	PRJNA360554	GCA_002082085.1	4.15622	56.4	MTBI01	9	3707	09/04/2017	Contig
*Alcaligenes faecalis subsp*. *faecalis*	NCTC10388	SAMEA104200667	PRJEB6403	GCA_900445215.1	4.24524	56.3	UFSQ01	8	3756	05/08/2018	Contig
*Alcaligenes faecalis*	MOR02	SAMN02997353	PRJNA258399	GCA_000770015.1	4.40271	56.4	JQCV01	23	3976	29/10/2014	Contig
*Alcaligenes faecalis*	AF_174	SAMN10249203	PRJNA497126	GCA_003939865.1	4.2815	56.5	RHXK01	16	3861	11/12/2018	Scaffold
*Alcaligenes faecalis*	GZAF3	SAMN06200346	PRJNA353361	GCA_002119995.1	4.34962	56.8	MSZP01	31	3885	08/05/2017	Contig
*Alcaligenes faecalis*	LK36	SAMN10134346	PRJNA506988	GCA_008373885.1	4.35931	57.0	RAQX01	50	3918	11/09/2019	Contig
*Alcaligenes faecalis subsp*. *faecalis NCIB 8687*	NCIB 8687	SAMN00998594	PRJNA86069	GCA_000275465.1	3.89962	57.2	AKMR01	186	3433	27/06/2012	Contig
*Alcaligenes faecalis*	NBIB-017	SAMN04230916	PRJNA300936	GCA_001530325.1	4.16548	56.4	LNOL01	17	3715	21/01/2016	Scaffold
*Alcaligenes faecalis*	GZAF5	SAMN06200348	PRJNA353361	GCA_002120045.1	4.44137	56.9	MSZR01	58	3989	08/05/2017	Contig
*Alcaligenes faecalis*	GZAF4	SAMN06200347	PRJNA353361	GCA_002120025.1	4.44353	56.9	MSZQ01	71	3985	08/05/2017	Contig
*Alcaligenes faecalis*	GZAF1	SAMN06200344	PRJNA353361	GCA_002120075.1	4.38790	56.8	MSZN01	61	3966	08/05/2017	Contig
*Alcaligenes faecalis*	GZAF2	SAMN06200345	PRJNA353361	GCA_002119985.1	4.39058	56.8	MSZO01	72	3969	08/05/2017	Contig
*Alcaligenes faecalis subsp*. *phenolicus*	IITR89	SAMN04371300	PRJNA307081	GCA_001516865.1	3.77406	57.6	LQAS01	23	3282	14/01/2016	Scaffold
*Alcaligenes faecalis*	UBA11281	SAMN08019624	PRJNA417962	GCA_003521065.1	3.92469	55.4	DOTO01	11	3526	09/09/2018	Scaffold
*Alcaligenes faecalis*	UBA7622	SAMN06456063	PRJNA348753	GCA_002484125.1	4.16785	56.9	DLJG01	15	0	05/10/2017	Scaffold
*Alcaligenes faecalis*	UBA7629	SAMN06451637	PRJNA348753	GCA_002484005.1	3.92651	55.9	DLIZ01	9	0	05/10/2017	Scaffold
*Alcaligenes faecalis subsp*. *phenolicus*	DSM 16503	SAMN02441201	PRJNA185539	GCA_000429385.1	4.24831	56.4	AUBT01	27	0	14/07/2013	Scaffold
*Alcaligenes faecalis*	UBA7605	SAMN06451956	PRJNA348753	GCA_002476455.1	3.99118	56.4	DLJX01	8	0	05/10/2017	Scaffold
*Alcaligenes faecalis*	UBA3878	SAMN06452818	PRJNA348753	GCA_002392125.1	3.68425	57.2	DGGI01	16	0	27/09/2017	Scaffold
*Alcaligenes faecalis*	UBA3227	SAMN06452319	PRJNA348753	GCA_002362965.1	4.04569	56.0	DEVV01	11	0	25/09/2017	Scaffold
*Alcaligenes faecalis*	UBA10732	SAMN08019755	PRJNA417962	GCA_003511485.1	3.98303	55.9	DOIE01	24	3600	06/09/2018	Scaffold
*Alcaligenes faecalis*	UBA8757	SAMN08018838	PRJNA417962	GCA_003507095.1	4.07836	55.0	DNWO01	307	4130	06/09/2018	Scaffold
*Alcaligenes aquatilis*	QD168	SAMN09984971	PRJNA489687	GCA_003671915.1	4.32388	56.3	chr: NZ_CP032153.1	1	3.815	22/10/2018	Complete

### Genome map and genomic islands prediction

The Mc250 genome map was constructed using Artemis DNAPlotter [26] and BRIG [27]. Genomic islands and phage regions were predicted according to Oliveira Alvarenga et al. [28].

### FliC phylogenetics and 3D structure prediction

The evolutionary history of the *fliC* gene was investigated using PSI-Blast [29] search with a hit limit of 1,000 accessions. Multiple alignment of the protein sequences was done using Muscle [30]. Model selection, gene tree Maximum Likelihood estimation, and branch support (by UFBoot) were all performed using IQTree2 [31]. The tridimensional structure prediction of the FliC protein was done using the Phyre 2 program [32].

### Pan and core genome analysis

Pan and core genome analysis was done with Roary [33] with a protein identity threshold of 90% [25, 33].

### *In silico* metabolic pathways comparison

All comparative analyses involving metabolic pathways and cellular processes were done within the RAST platform [34].

### *In vitro Xanthomonas citri* antagonistic assay

*In vitro* assays were performed by inoculation of Mc250 over a lawn of *Xanthomonas citri* subsp. *citri* strain 306 pathotype A (Xac306) previously made in LB agar plate (90 × 15 mm Petri dish). Halo formation indicated inhibition capacity after 2 days at 28°C. *Serratia marcescens* and *Escherichia coli* were used, respectively, as positive and negative controls. For the formation of the *Xanthomonas* lawn, 20 μL of a culture in liquid LB medium with OD = 1 was applied onto the surface of the medium and spread homogeneously with a Drigalski handle.

### *In vivo Xanthomonas citri* antagonistic assay

*In planta* assays to evaluate Mc250 antagonism to Xac306 were carried out by their co-inoculation in sweet orange grafted plants (*Citrus sinensis* (L.) Osbeck. “Pera Rio”). Twelve-month-old plants were kept in a growth chamber at 28°C and under a photoperiod of 16 h. Mc250 were inoculated together with Xac306 in the abaxial region of citrus leaves under infiltration pressure 1 mL with needleless syringes. The final concentration of Xac306 and Mc250 suspensions were adjusted to 10^7^ CFU/ml in 10 mM MgCl_2_. Plant inoculations with Xac306 alone or MgCl_2_ were used, respectively, as positive and negative controls. The infiltrated leaves were photographed 3 and 14 days after inoculation (DAI). The lesions area of five infiltrated leaves (from three independent assays) were quantified, and infected areas were calculated using Image J v1.48 [35].

### Phytopathogenic nematodes mortality assay

Mc250 was cultured in LB medium at 28°C on a shaker (180 rpm) for 48 h. After this time the bacterial culture was centrifuged at 10000 x*g* for 10 min and the supernatant was used in the following experiments. To determine the effect of Mc250 extracellular metabolites on the nematodes *Pratylenchus brachyurus* and *Panagrellus redivivus*, 100 μL of the culture supernatant was transferred to multi well plate previously loaded with a suspension of 100 μL per well containing 100 nematodes of each species previously axenized. The plate was kept at 28°C for 48 h and nematode mortality was evaluated according to the methodology described by Chen and Dickson [36]. A negative control was performed by replacing the culture supernatant by the same volume of sterile LB medium. The assay was conducted in a randomized design with five replicates per treatment.

### Statistical analyses

Statistical analyses were performed using the statistical package GraphPad Prism version 5.00™ (San Diego, CA). The results were submitted to the normality test of Smirnov Kolmogorov and represented as the mean ± SEM (standard error of mean) or mean ± SD (standard deviation). The Student's t-test was used to compare pairs of parametric groups while variance analyses one-way ANOVA was used to compare three or more groups with Tukey post tests for parametric data, while Kruskal-Wallis test was used to compare Dunn's posttests data, considering *p* <0.05 (*), *p* <0.01 (**), and for *p* <0.001 (***).

## Results

### General characteristics of Mc250 genome

The *Alcaligenes faecalis* strain Mc250 (Mc250) genome was sequenced using the Illumina MiSeq platform, resulting in 5,867,947 paired-end reads, which were assembled into one circular contig. The Mc250 chromosome has 4,159,911 bp; its automatic annotation resulted in 3,719 protein coding genes, 26 pseudogenes, 57 tRNA sequences, three rRNA operons, four ncRNA genes, and no CRISPR array. No plasmid was identified.

### Phylogenomics and pan- and core genome analysis

**Fig 1:**
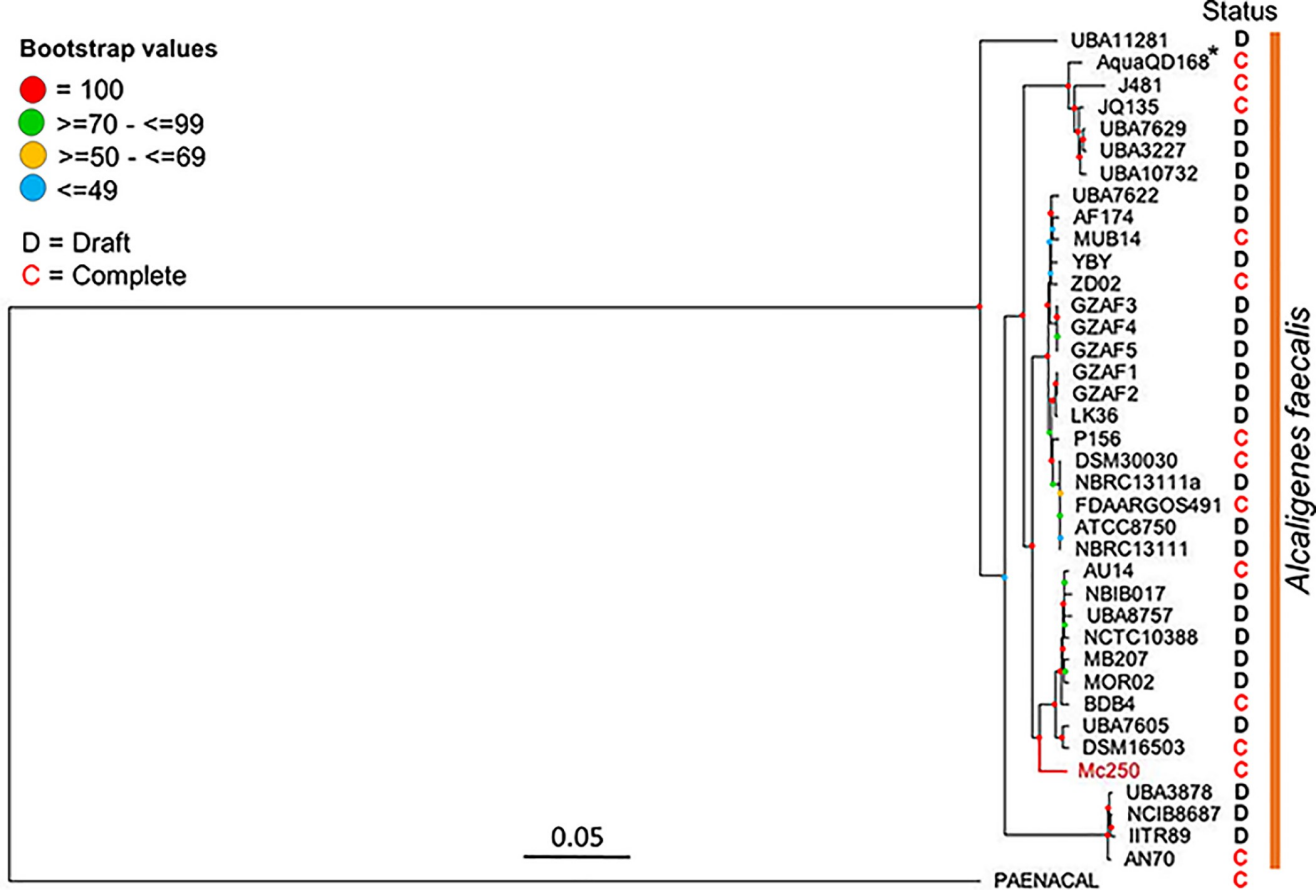
Phylogenenomic analysis of 37 *Alcaligenes faecalis* strains. Bootstrap values are represented according to the placement of the circles (see legend in the figure). *A*. *quatilis* strain QD168 (*) and *Paenalcaligens hominis* were incorporated respectively as a member belonging to a distinct species and as a group outside the genus *Alcaligenes* (from the same family Alcaligenaceae); the genomes of both are complete. Status: D–Draft and C–complete.

A Maximum Likelihood (ML) tree showed that Mc250 clusters within others genomes of the *Alcaligenes* genus, and particularly within one of the *A*. *faecalis* clades (Fig 1), hence confirming its classification.

For pan- and core genome analysis purposes we compared Mc250 with thirty-six other *Alcaligenes faecalis* genomes available as of March, 2020 (Table 1). This analysis showed that the pan-genome has just under 11,000 genes, whereas the core genome has 1,459 genes, or about 39% of the complement of protein-coding genes in Mc250 (S1A Fig). The pan-genome curve is clearly ascending (S1B Fig). Both of these results show that there is substantial variation in gene content among known *A*. *faecalis* genomes. The Mc250 genome contains 250 specific genes with respect to the other genomes, at the 90% identity threshold (S1 Table).

### Genome islands

**Fig 2:**
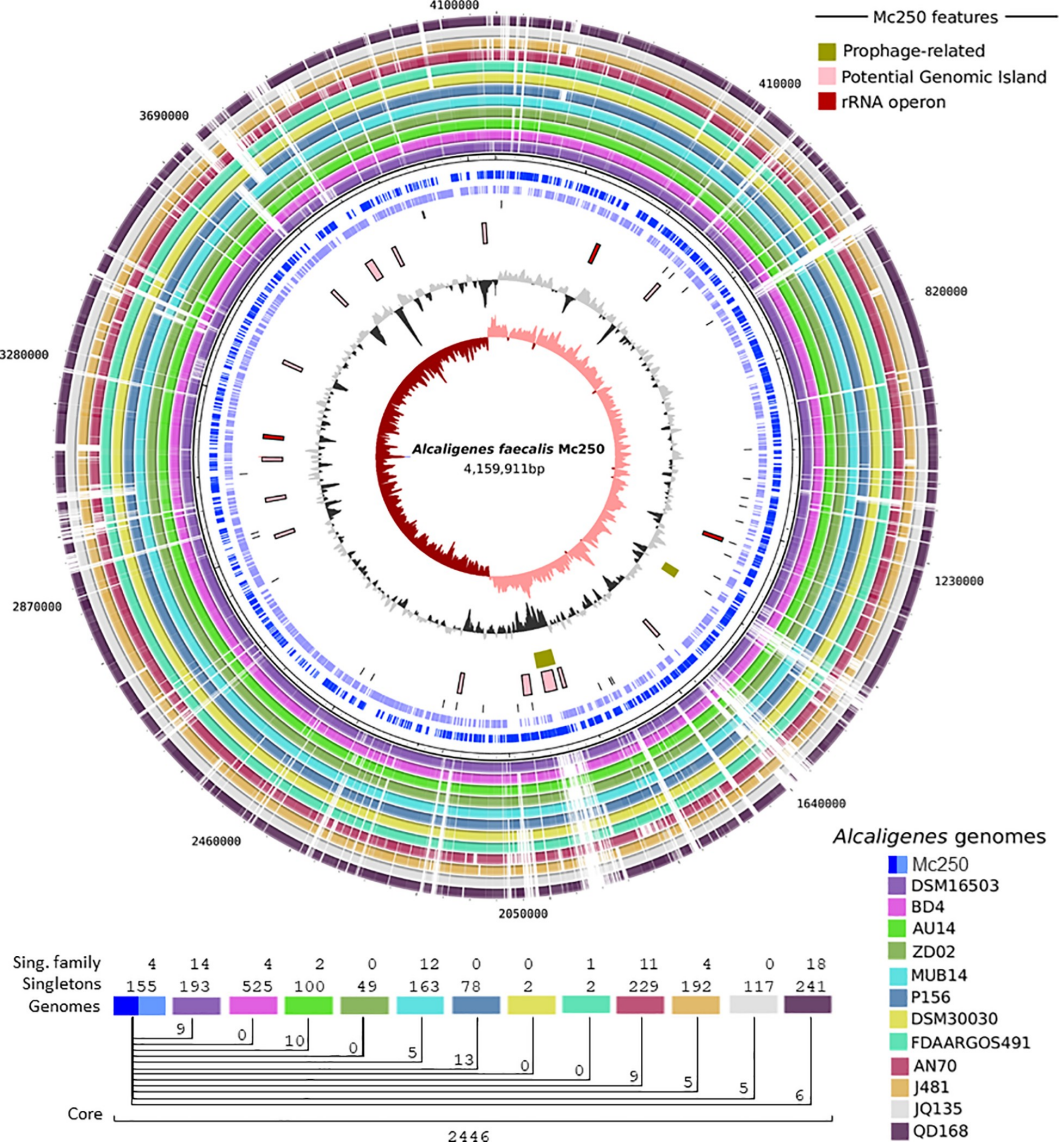
Mc250 circular genome. (A) The 12 outermost circles denote orthologous genes present and absent in the respective organisms identified in the legend of the figure. The first two inner circles highlight the location of the Mc250 genes positioned on the + (dark blue) and—(light blue) bands. The 3^rd^ inner circle identifies the positions of tRNAs. The 4^th^ and 5^th^ inner circles identify potential genomic islands and rRNA operons and prophage related regions, respectively. 6^th^ inner circle—GC content (GC%). 7^th^ inner circle—GC cumulative indicating in salmon positive values and negative values in dark red. (B) Presence of unique genes (singletons) and shared between the analyzed only among the complete genomes analyzed. The colors that identify the genomes are those used for identification in the circular genome.

When compared to 36 *A*. *faecalis* genomes (Table 1), a total of 14 genomic islands and two prophage regions were identified in Mc250 (Fig 2A). These regions have horizontal gene transfer features such as strong deviations in GC content from the rest of the genome, presence of integrases or recombinases, and in the case of both prophage-like regions, the presence of flanking tRNAs, suggesting the integration of a temperate bacteriophage. Moreover, most of these regions are located in areas where there is no alignment to other *Af* genomes (S2 Table and Fig 2A), with a total of 155 genes found only in the Mc250 genome (Fig 2B). A complete list of the genes found in these regions is presented in S2 Table.

### Functional analysis of the Mc250 genome

We now present results of a functional analysis of the Mc250 genome. In these analyses we compared the Mc250 genome with the genomes of twelve other *A*. *faecalis* genomes (those that are complete and considered clade representatives based on the phylogeny we obtained (Fig 1)). The information associated with the locus tag and metabolic functions of the genes described in this section are present in the S3 Table.

### Metabolism of phenolic compounds

**Fig 3:**
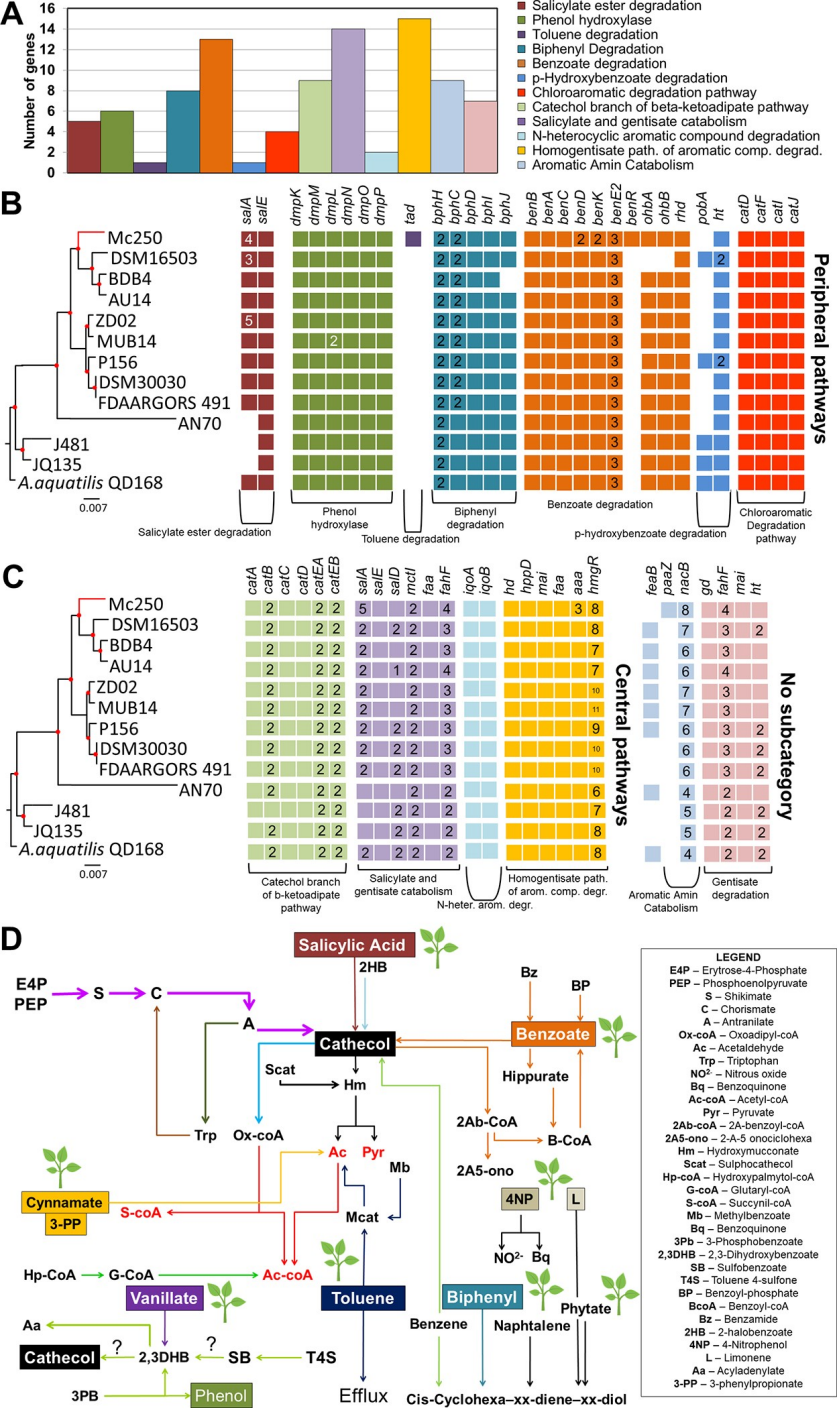
Metabolism of phenolic compounds. (A) Representation of genes associated with thirteen metabolic pathways involved with degradation of phenolic compounds, using RAST [37]. (B, C) Comparative analysis of 94 genes associated with degradation of phenolic compounds present in the genome of Mc250 with respect to 12 other related genomes. (D) Metabolism reconstruction of degradation pathways of phenolic compounds in Mc250. The colors of the substrates and pathways represent the same colors as those depicted in (A). Most pathways converge to the synthesis of pyruvate, acetyl co-A or succinyl-coA, all intermediates of glycolysis or TCA. The symbol of plants highlight that the compound is produced in plants as mechanisms of defense against pathogens. Information on the genes shown in this Figure is available in S3 Table.

In order to determine what Mc250 genes could help explain its survival capability in contact with the roots of *Mimosa calodendrom*, a comparative analysis of metabolic pathways involved in phenolic compound degradation was performed. Of the 3,719 protein-coding genes, 94 (2.5%) were categorized into 12 metabolic pathways associated with degradation of these compounds (Fig 3A). We investigated the presence of the genes in these pathways in 12 other related genomes (Fig 3B and 3C). An integrative analysis of the relationship between these pathways was carried out (Fig 3D).

### Resistance to antibiotics and heavy metals

The Mc250 genome has several genes associated with antibiotic resistance. Six genes associated with multidrug efflux pumps (*cmeAB*, *tolC*, *mdr*, *macAB*, *oml* and *acrB*) were identified, ranging from a single copy to six-copy paralogs (S2A Fig). Genes associated with resistance to fluoroquinolones (*parCE* and *gyrAB*) were also identified. A repertoire of genes associated with detoxification and metabolism of copper, arsenic, iron, cobalt, and zinc (S2B Fig) was found. The *arsRBCH* gene cluster, which encodes a transcript regulator, a transporter, and an arsenic resistance gene, respectively, involved arsenate detoxification [38], was identified in genomic island 10.

### Iron acquisition and metabolism

We found 12 genes associated with siderophore biosynthesis. These genes are: *ybdZ*, and immediately downstream, *entCEBA*, followed by *entS*, the gene that encodes a siderophore carrier protein; *fes*, a gene encoding for enterobactin esterase; *entF* (synthesis component, serine activating enzyme); and the transport system of this compound to the medium (*fepAGDCB*) (S2B Fig). Eleven genes associated with iron acquisition were also found. Among them four copies of *pitADC* genes (two of which are complete and in tandem), which correspond respectively to subunits of iron-binding, ATP-binding, and permease proteins of an ABC transporter system. We also found the ABC transport system of ferrichrome/iron (III) dicitrate (*fhu/fec*), plus a gene for the receptor for hemin (*hemR*), two copies of the *tonB* gene, which codes for a periplasmic protein involved with transport of iron-chelated siderophores, a gene coding for a protein that utilizes heme groups (*hutX*), and two genes coding for paraquat-inducible proteins (*parAB*).

### Stress response

We found 138 protein-coding genes (3.7%) associated with some type of stress response. Of these, 23 were associated with osmotic stress, including: *osmB*, *osmY* (Osmotically inducible lipoprotein), *yciM* (heat shock protein), aquaporin Z, five genes associated with ectoine synthesis and regulation (*ectRABCD–*described in detail below), and 11 genes associated with choline and betaine uptake, including *betA*, *betB*, *betT*, *betC*, *sox*, *gbcA* and two copies of the cluster encoding the ABC transporter *proU* (S3A Fig). Another 16 genes were annotated as heat shock-associated, including the cluster formed by *dnaJK-grpE-hrcA*. Finally, the genes *cspC* and *cspD* were annotated as associated with cold shock response, and seven other genes (*degS*, *rseA*, *skp*, *degP*, *rseB*, and *surA* (two copies)) annotated as associated with periplasmic stress response. Sixty-two genes related to oxidative stress protection were found (S3A Fig), including: the regulatory genes *fur* (two copies), *zur*, *soxR* and *fnr* (three copies), *sodA* [Mn], *sodB* [Fe], *sodC* [Cu-Zn], catalase HPII, *ahpC*, *dps*, and glutaredoxin (*grx1*), six genes associated with glutathione biosynthesis (*gshA*, *gshB*, *hyp1*, and *gltT* (three copies)), and sixteen other genes associated with non-redox reactions (*sam1-gloB*, *yfcF* (two copies), *gloA* (three copies) and nine copies of gluthatione transferase encode genes).

### DNA and RNA metabolism

The Mc250 genome has 95 genes (2.5%) associated with DNA metabolism, of which 71 are involved with DNA repair. Among these, we highlight *mutS*, *mutL*, *uvrA*, *uvrB*, *uvrC* and dimeric *uvrA*, *uvrD*, a gene coding for photolyase, *recA* and *recX*, nine genes associated with the RecFOR pathway (*recO*, *recR*, *recQ*, *recA*, *recA* and four copies of *ssb*), nine genes associated with base excision repair, and two helicases (S3B Fig). As for RNA metabolism, 162 genes (4.3%) were annotated with this function, of which 24 are associated with transcription and 142 associated with RNA processing and modification. In this category, we mention nine genes associated with tRNA modification at position N34 as well as 31 genes associated with queuosine-archeosine biosynthesis, of which 21 are copies of genes coding for permease of drug/metabolite transporter (DMT).

### Secondary metabolites production

**Fig 4:**
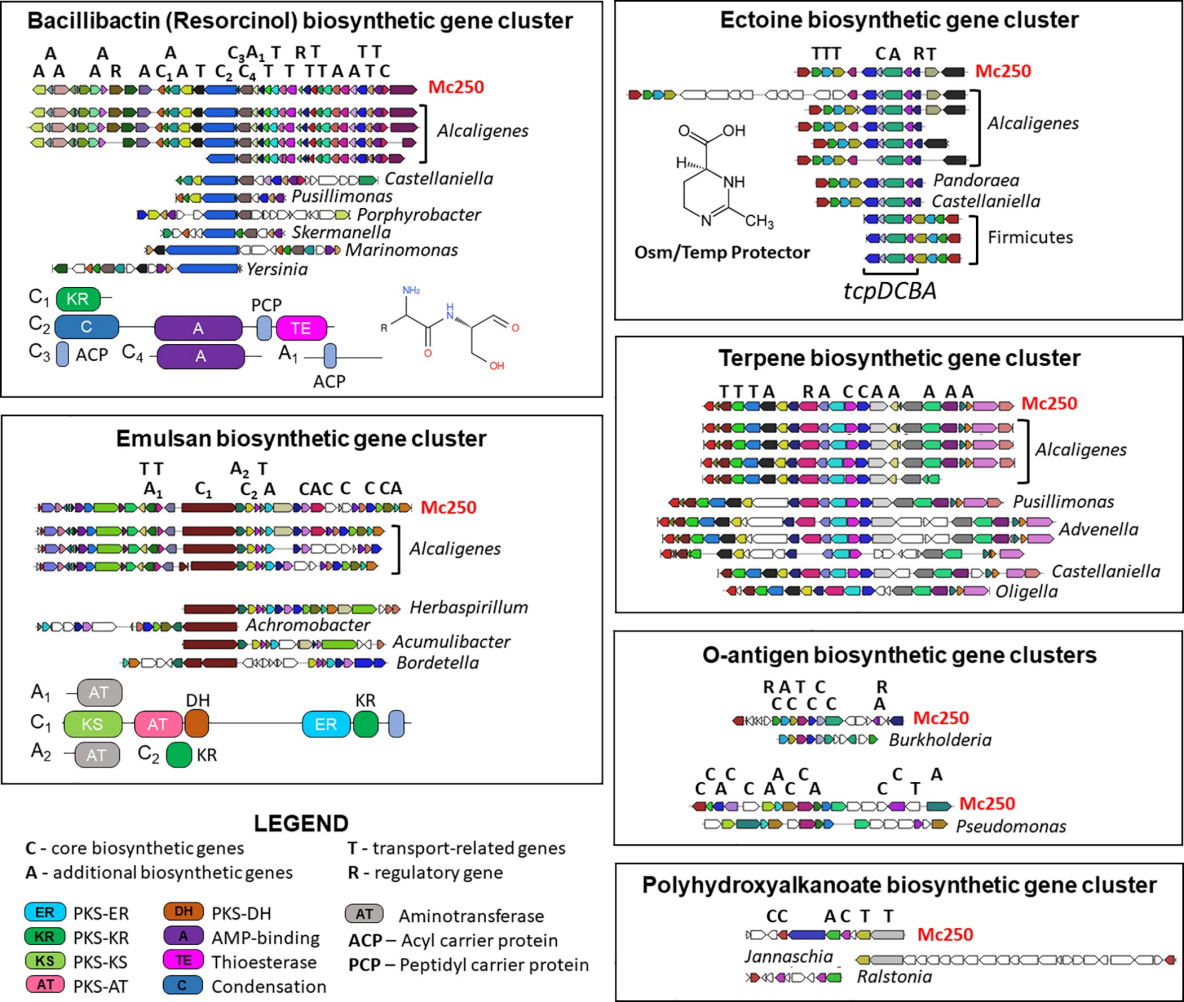
Characterization of the gene cluster associated with synthesis of secondary metabolites in the Mc250 genome. The small arrows denote the genes related to a specific biosynthetic cluster, whose colors are associated with their respective functions. The letters A, R, T, and C identify the functional characteristics of the genes in the metabolic synthesis process, as detailed in the legend. The domain structure of a few core (C) and additional (A) biosynthetic genes are provided in the resorcinol and emulsan panels (with symbols explained in the figure legend). For all regions, the reference genome is Mc250. Details of these clusters are shown in S4 Table.

Secondary metabolites produced by Mc250 were predicted by AntiSmash 3.0 [39] (S4 Table and Fig 4). Among the 18 gene sets identified, 11 were characterized with Cf-putatives; one cluster associated with Bacillibactin synthesis; one cluster associated with synthesis of T1pks-Cf_saccharide involved with emulsan biosynthesis; one cluster associated with ectoine biosynthesis; one cluster associated with terpene synthesis; two clusters associated with O-antigen synthesis; and one cluster associated with polyhydroxyalkanoate biosynthesis.

### Sulfur metabolism

In the Mc250 genome, 75 genes (2%) associated with sulfur metabolism were identified, of which 23 are associated with inorganic sulfur assimilation and 34 with assimilation and use of organic sulfur. Among these34 genes, we mention the genes associated with uptake and use of taurine and alkanesulphonates (S4A and S4B Fig). Integrative analysis of these pathways suggests that Mc250 exhibits high cysteine synthesis capability as a byproduct of the sulfur metabolism (S4B Fig). In addition to being used as a key amino acid for protein synthesis, it is possible that the cysteine surplus produced is used in the synthesis of molecules associated with protection against oxidative stress, as in the case of glutathione (S4C Fig).

### Potassium homeostasis

The Mc250 genome codes for several genes related to the influx and efflux of potassium. We found genes coding for the ABC transporters of the Kdp system (*kdpFACB*) and their regulatory genes, coding for the sensory and regulatory proteins of this system (*kdpED*) (S4D Fig), all arranged in tandem. In addition, a copy of the *kup* (low-affinity potassium transport system) gene, two copies of the *trkA* gene (TrkA system potassium uptake protein) and two copies of the *kefA* (potassium efflux system KefA) gene were also found. Additionally, we found two copies of a gene coding for the glutathione-regulated potassium efflux system ATP binding protein (*kefBC*).

### Secretion systems

No genes coding for proteins of the T3SS, T5SS, T7SS and T8SS secretory systems could be found. However, eleven genes associated with the T1SS were found (S3E Fig). We found two copies of the genes encoding the *lapBCD* apparatus, with an additional copy of *lapD*, and four copies of the gene encoding RTX-like adhesins, one of which is located in tandem with one of the *labBCD* clusters. The Mc250 genome has eleven genes coding for the general secretion pathway of the T2SS (*gspGHIJKLMNDEF*) arranged in tandem. Fifteen T6SS genes were found, twelve of which were in tandem *impABCDFGH-vasDJK-icmF-impM* in addition to *vgrG* (sigma-54 dependent transcriptional regulator), *clpB* (chaperone protein), and *vasH*. Genes coding for the twin-arginine translocation (*tat*) system were also found.

### Membrane proteins

In addition to the presence of four secretory systems, we identified 52 genes (1.4%) associated with ABC transporters: five genes associated with oligopeptide transport (*opp genes*), 37 genes associated with branched amino acid transport (*livJHMGF*) distributed in nine gene clusters, and 10 other genes associated with dipeptide transport (*ddpABCDF*). We also identified 56 genes (1.5%) associated with TRAP transports. We also found 17 genes associated with tricarboxylate transport, two adjacent copies of *tctAB* genes, ten copies of *tctC*, and two copies of *tctD*. Finally, we found two complete copies of the *tonB-exbBD* cluster, two tandem copies of *exbBD*, and a cluster formed by the *trp-pal-tolBA-htaS*. Regarding membrane proteins involved with metal transport, we found *mgtC* (Mg^2+^ + transport ATPase type C), two copies of the *mgtE* (Mg/Co/Ni transport protein), three copies of the *corC* (Mg/Co efflux protein), and four copies of *corA* (Mg/Co transport protein), three of which are adjacent, and three copies of *copA* (copper effux protein); the products of these genes are associated with magnesium, cobalt, nickel, or copper transport.

### Motility and chemotaxis

Genes associated with biosynthesis (45 genes) and regulation of flagellum activity (18) were found. Although no gene associated with fimbria synthesis was found, eleven genes associated with the *tad* locus were found, arranged in two clusters (Planet et al. refer to this gene set as the Widespread Colonization Island [40]) A single gene encoding the Flp pilus assembly protein was found.

### *FliC* gene analysis

FliC has been described as an important Microbe-Associated Molecular Pattern (MAMP) protein [41], and when present in pathogenic organisms, it has also been described as a Pathogen-Associated Molecular Pattern (PAMP) protein, capable of modulating defense responses in animal and plant hosts [42, 43]. In this context, FliC is directly associated with an intricate bacterial-host interaction system [44].

**Fig 5:**
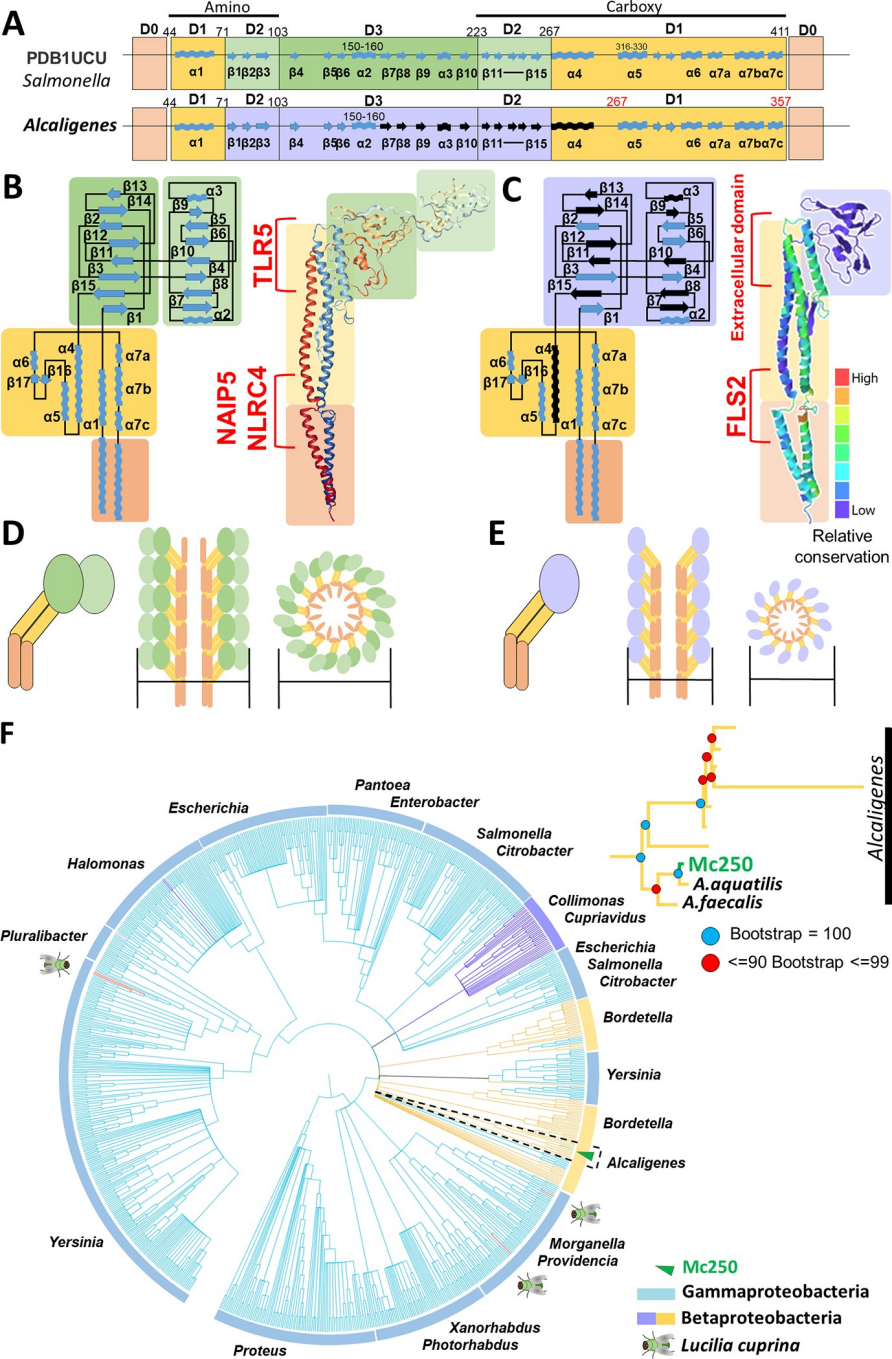
Structural analysis of the fliC genes and their corresponding proteins in a model organism (*Salmonella typhymurium* strains LT2, StLT2) and Mc250. (A) Organization of *fliC* domains found in the model protein (PDB1UCU) compared to Mc250. D1 (yellow), D2 (light green), D3 (dark green) and D0 (orange) represent terminal domains in flagellins, adapted from Yonekura, Maki-Yonekura and Namba [45]. The secondary structures identified by the black color in the Mc250 sequence correspond to the loss of 130 residues of the sequences of all *Alcaligenes* species when compared to bacteria of other genera. (B) Schematic representation of the secondary structural domains in FliC of StLT2 (PDB1UCU) adapted from Song et al. [46]. (C) Schematic representation of secondary structural domains of FliC in Mc250. The colors from red to blue show the relative degree of conservation of the amino acid residues. (D) Simplified model of the FliC conformation of StLT2 in the monomeric form, and polymeric in the constitution of the flagellum (lateral and superior view). (E) Simplified model of the FliC conformation of Mc250 in the monomeric form, and polymeric in the constitution of the flagellum (lateral and superior view). NAIPG, NLRC, and TLR5 identify the binding sites of specific antibodies and receptors in animal hosts. FLS2 and extracellular domain identify the binding sites of plant receptors. (F) FliC ML gene tree based on PSI-Blast. The group of *Alcaligenes* constitute an isolated clade identified by dashed lines. Within this clade (see zoomed-in section to the right), Mc250 is in a subgroup together with *A*. *aquatilis* and another strain of *A*. *faecalis*.

A multiple alignment of the sequence of *fliC* from Mc250 with similar sequences from other bacteria of the genus *Alcaligenes* retrieved from NCBI by a BLAST search showed a wide variation in the composition of residues located between positions 161 and 280 (Fig 5A). In contrast, the amino and carboxy terminal regions in this alignment have high similarity levels (98 and 100%, respectively). In addition, we observed that the length of the FliC proteins in this alignment (mean of 357 residues) was substantially shorter than the length of orthologs in other genera (mean of 490 residues). Multiple alignment of the Mc250 FliC sequence with sequences from orthologs in non-*Alcaligenes* bacteria revealed that the missing 133 residues are located in a position immediately preceding the variable region described above. An analysis based on 3D models suggests that the flagellins in bacteria of the genus *Alcaligenes* do not have the secondary structure corresponding to secondary structures from β7 to α4, which corresponds to a partial loss of the D3 and D1 domains, and a total loss of the D2 domain (Fig 5A). As D2 and D3 are domains present on the external face of the flagellum, after polymerization of FliC [45], it is possible that the thickness of the flagella in these bacteria is smaller, as previously described in other bacteria, such as *Salmonella* (Fig 5B–5E).

This result prompted us to investigate the evolutionary history of *fliC*. A *fliC* maximum likelihood tree (Fig 5F) shows Betaproteobacteria homologs clustered in at least five different clades, three of them including only *Alcaligenaceae*, and the other two having non-*Alcaligenaceae* genomes only. Four homolog copies belonging to the dipteran insect *Lucilia cuprina* were also found by PSI-Blast, spread in three different positions across the tree; all four belong to a WGS accession (NW_019410486.1) of *Lucilia cuprina* strain LS. Three homologs are located in scaffold 12 in the available *Lucilia cuprina* genome assembly (LOC111676276, LOC111676277, and LOC111676345), whereas the fourth copy is located in scaffold 1053 (LOC111686045).

### Plant-Mc250 interactions

In the next sections, we present results related to metabolic pathways and physiological systems inferred from the genome of Mc250 that may play a role in bacteria-plant interactions.

### Nitrogen metabolism

**Fig 6:**
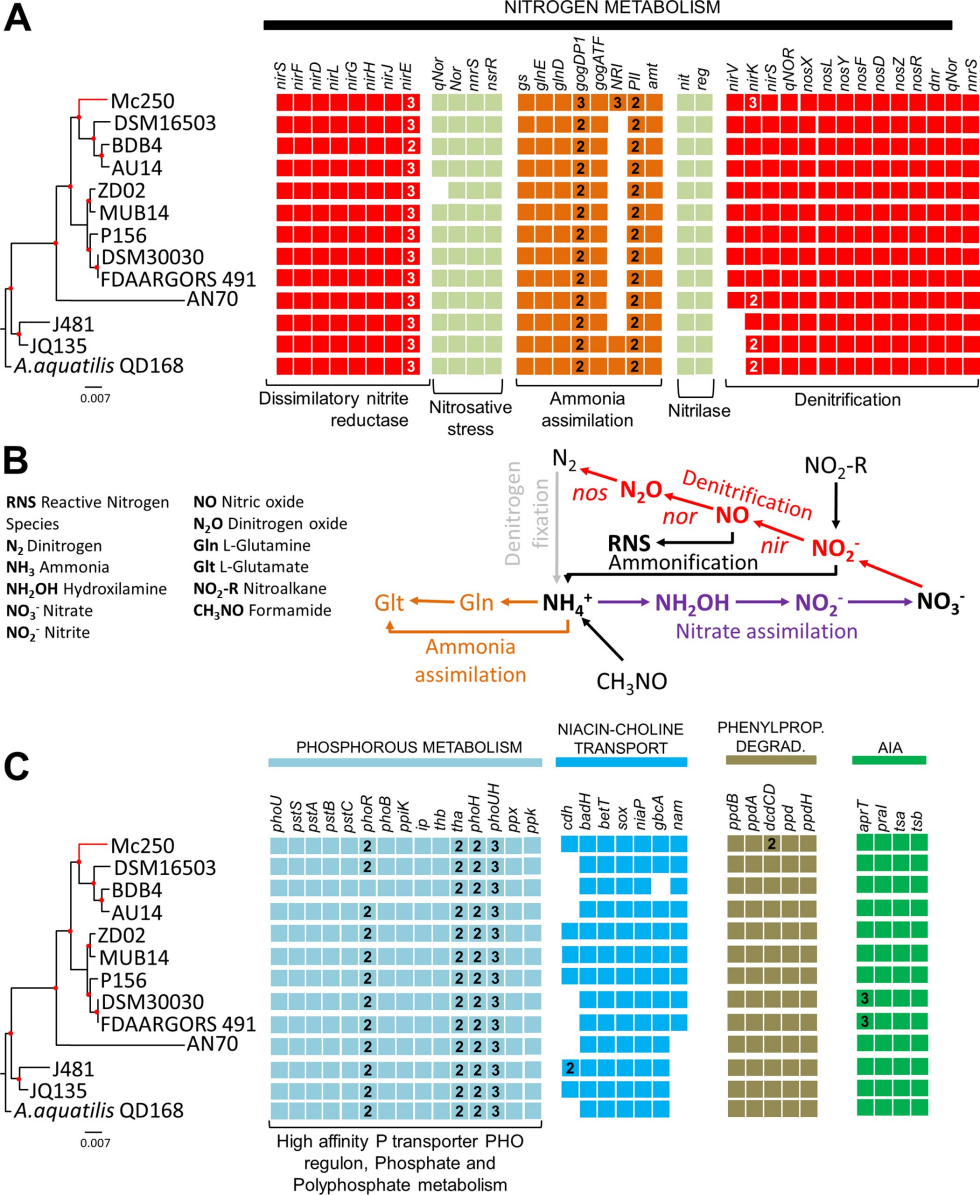
Identification and comparison of genes associated with plant-growth promotion. (A) Analysis of the genes associated with nitrogen metabolism in the Mc250 genome in relation to the other twelve Alcaligenes genomes investigated. (B) Nitrogen metabolism pathways. Most of the genes are associated with the denitrification pathway (in Red) (C) Analysis of the genes associated with siderophore production, phosphorus metabolism, and IAA synthesis in the Mc250 genome compared to the other twelve Alcaligenes genomes investigated.

Twenty-eight genes were found related to nitrogen metabolism (Fig 6A). Of these, ten (*nirECFDLGHJES*) are associated with dissimilatory nitrite reductase pathway. In addition, we also found 13 genes associated with the ammonia assimilation pathway, including *glnD* (Protein PII uridyltransferase), *glnE* (glutamate-ammonia-ligaseadenyltransferase), a gene coding for glutamine synthase type I, a gene coding for a ferrodoxin-dependent glutamate synthase, three copies of a gene coding for nitrogen regulation protein NR (I), two copies of a gene coding for nitrogen regulatory protein P-II (one of which is adjacent to the gene coding for ammonia transporter), two copies of a gene coding for the large subunit of a glutamate synthase (NADPH) (one of which is adjacent to the gene coding for the small subunit). We also found genes coding for a nitrite-sensitive transcriptional repressor (*nsrR*), a protein involved in response to NO (*nnrS*), and a quinol-dependent nitric oxide reductase (*qnoR*). We also found two copies of the gene coding for nitrilase, one associated with plant-induced nitrilase (*nit*) and the other associated with a transcriptional regulator adjacent to a plant-induced nitrilase gene (*reg*). We found 26 genes related to nitrification, seven of which form the cluster *nirXLYFDZR*, plus *nirV* (nitrite reductase accessory protein), three copies of *nirK* (Copper-containing nitrite reductase), *qNor* (Nitric- oxidase-dependent quinolone), *nirS* (cytochrome cd1 nitrite reductase), *nnrS* (involved in the response to NO), and *dnr* (Fig 6B).

### Phosphorus metabolism

Thirty-two genes associated with phosphorus metabolism were identified. Of these, nine are associated with a high affinity system by this semimetal system, including the *pstBACS* genes (Fig 6C). This system is regulated by *phoBR* genes, which encode for a dual regulatory component. In addition, we found the *phoU* gene, coding for a regulatory protein. Another 21 genes were categorized as being associated with polyphosphate metabolism, including *ppx* (exopolyphosphatase) and *ppk* (polyphosphate kinase). These two genes are adjacent to the *pstBACS* cluster.

### Glycerol-3-phosphate and C4-Dicarboxylates uptake and metabolism

Glycerol-3-phosphate (G3P) and C4-dicarboxylates (C4-C) are produced by plants as infectious response signaling molecules [47]. The Mc250 genome has two clusters of the *ugpABCE* genes encoding the ABC transporter associated with G3P internalization. In addition, genes coding for enzymes encoding glycerophosphoryl diester phosphodiesterase (*ugpD*), glycerol kinase (*glpK*), and glycerol-3-phosphate dehydrogenase (*glpZ*) (two copies) were also found. Regarding the metabolism of C4-C, such as malate, oxaloacetate, and succinate, the Mc250 genome has an ABC transporter and genes that regulate the expression of this system (*dctBD*, sensor and regulator).

### IAA, acetoin and butanediol biosynthesis

No genes capable of converting tryptophan to IAA were identified, but the four genes associated with conversion of anthranilate to tryptophan were identified (Fig 6C). Likewise, no gene associated with HCN synthesis was found, although a carrier protein of this compound, *cynX* (cyanate transport protein), was identified. The adaptation of Mc250 in the presence of this compound may be related to the presence of nitrilases (described above in the section on nitrogen metabolism), which can detoxify this compound by providing ammonia to the plant. Finally, although genes coding for major and minor subunits of acetolactate synthase protein were found, the gene coding for acetolactate decarboxylase was not, which suggests that Mc250 may not be able to synthesize acetoin and 2,3-butanediol.

### Niacin and choline transport and metabolism

Niacin and choline are byproducts of plant metabolism exuded by roots [48]. In the Mc250 genome we found seven genes associated with niacin and choline transport and metabolism: these are genes coding for choline dehydrogenase, betaine aldehyde dehydrogenase, high-affinity choline uptake protein (*betT*), niacin transporter (*niaP*), glycine betaine demethylase subunit (*gbcA*), nicotinamidase, and the alpha subunit of sarcosine oxidase (Fig 6C).

### Phephylpropionate degradation

Six genes in this pathway were found: 3-phenylpropionate dioxygenase, alpha and beta subunits, 3-phenylpropionate dioxygenase ferredoxin-NAD (+) reductase component, 2,3-dihydroxy-2, 3-dihydro-phenylpropionate dehydrogenase and two copies of 1,2-dihydroxycyclohexa-3,5-diene-1-carboxylate dehydrogenase (Fig 6C).

### Mc250 nematicide and bactericide potential

**Fig 7:**
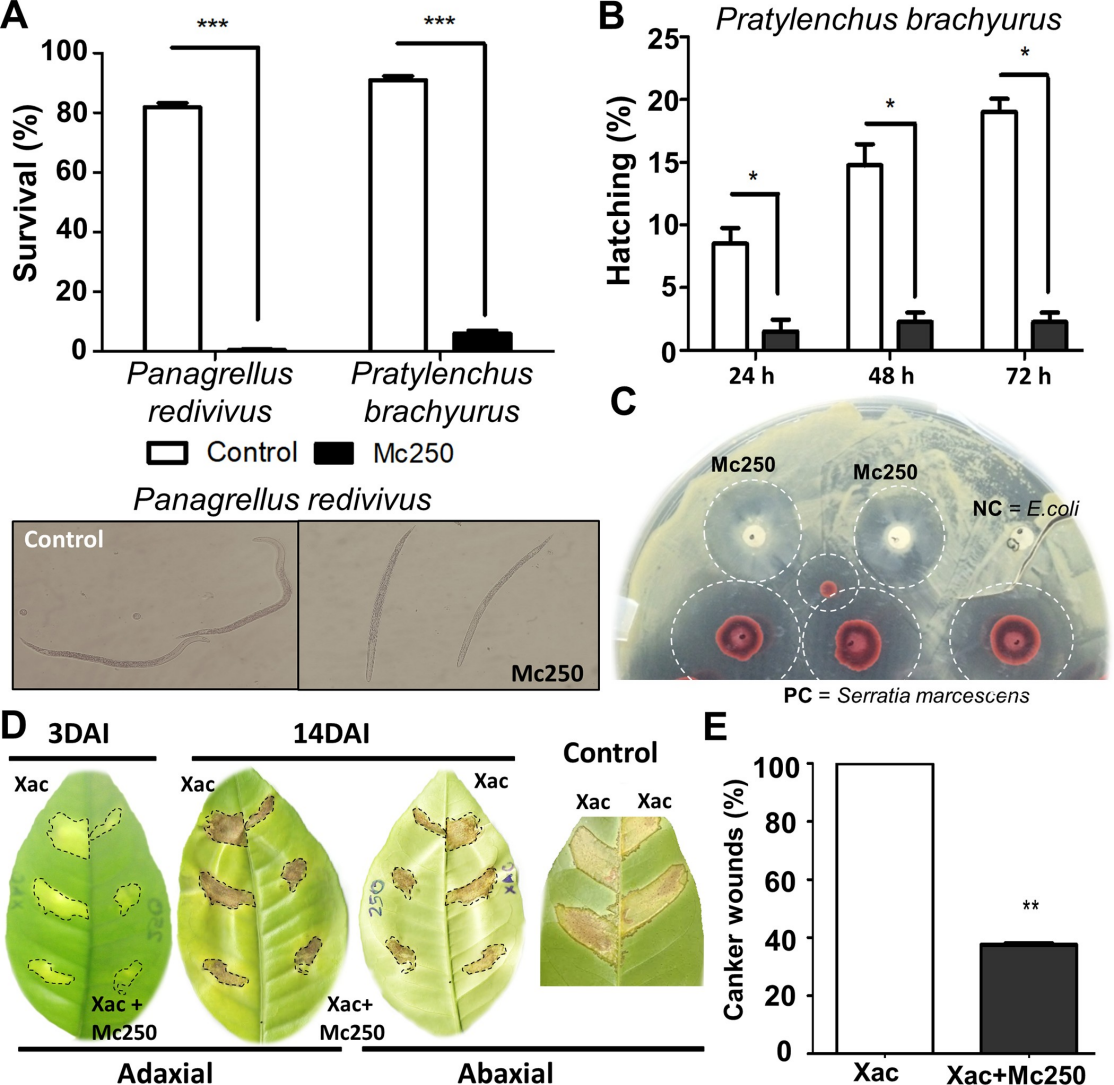
Anti-phytopathogenic effect of Mc250. (A) Analysis of the Mc250 inhibition potential against the juvenile nematodes of the genus *Panagrellus redivivus* and *Pratylenchus brachyurus*. (B) Analysis of the Mc250 inhibition potential against *Panagrellus redivivus* egg hatching. (C) Analysis of the Mc250 inhibition potential against *Xanthomonas citri* subsp. *citri* A306 *in vitro*. PC–Positive control (*Serratia marcescens*). NC–negative control (*Escherichia coli*). (D) Analysis of the Mc250 inhibition potential against A306 when co-inoculated with Mc250 in plants of *Citrus sinensis*. DAI–Days after innoculation. *: p <0.05; **: p <0.01; ***: p <0.001.

We investigated the ability of Mc250 to act as a biocontrol agent of phytopathogenic nematodes and bacteria. When in contact with juvenile nematodes of the species *Panagrellus redivivus* and *Pratylenchus brachyurus*, AfMc50 was able to kill 100% and 95% of the individuals, respectively, after 24 hours of contact (Fig 7A). Mc250 was also able to massively inhibit these species’ egg hatching after 24 hours of contact (Fig 7B). In addition, Mc250 was able to reduce the growth of *Xanthomonas citri* subsp. *citri* A306 *in vitro* (Fig 7C) and *in vivo* when co-inoculated with A306 in *Citrus* plants (Fig 7D), decreasing canker lesions by about 60% (Fig 6E).

## Discussion

The phylogenetic analyses of the Mc250 genome show that this strain belongs to the species *Alcaligenes faecalis*. The pan-core genome analysis also showed that there is large variation in gene content among the 37 *A*. *faecalis* genomes investigated; its “cloud genome” (S1A Fig) corresponds to more than half of the pan-genome. These results suggest that novel strains of *A*. *faecalis*, such as Mc250, can be an important source of new knowledge for the genomics of this versatile species.

**Fig 8:**
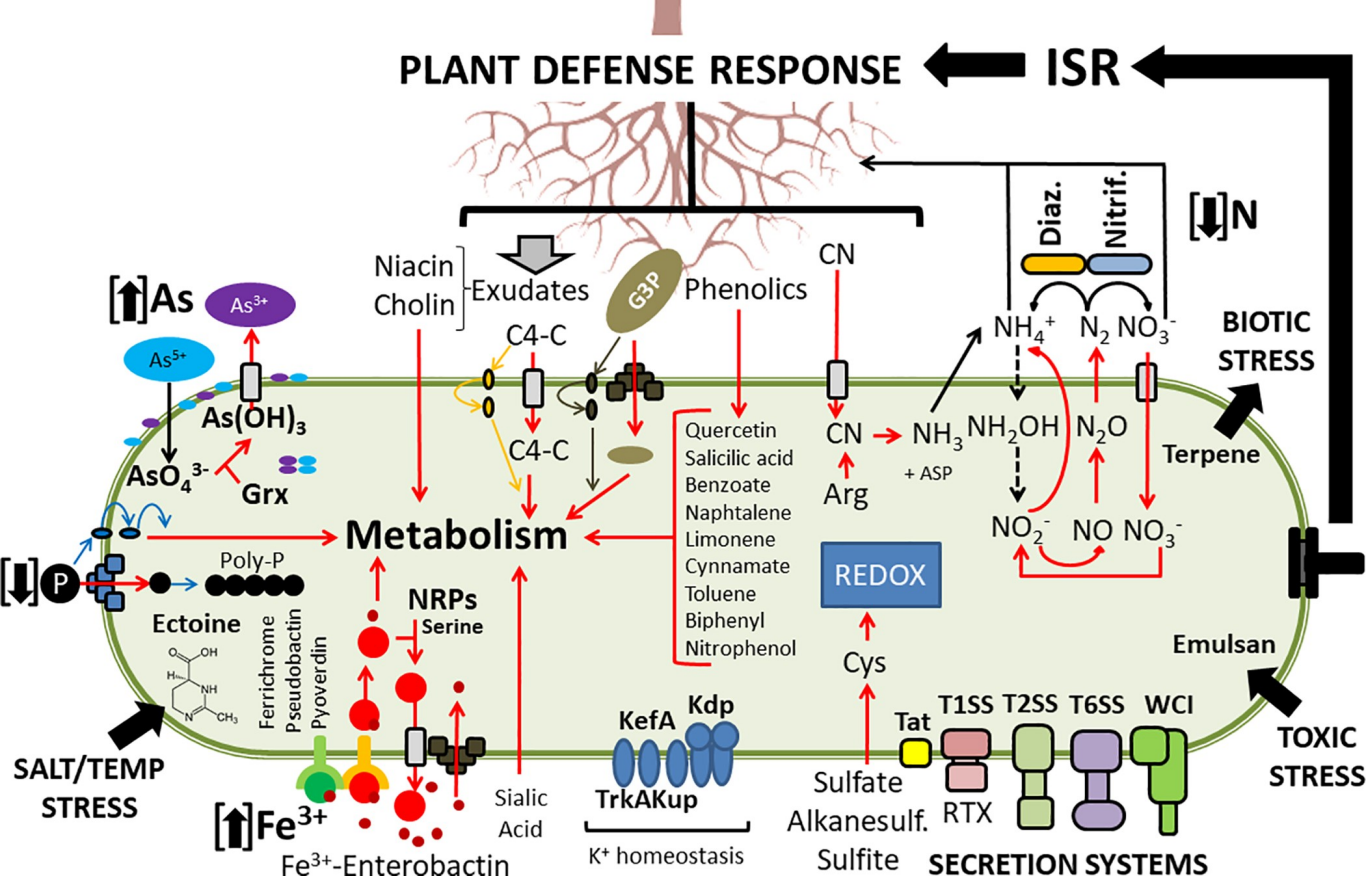
Integrated analysis of the metabolism of Mc250. This figure puts together the various metabolic inferences made based on the genome analysis. The red arrows denote the metabolic flow of pathways associated with adaptation to the environment or integration with plant metabolism. The dashed black arrows denote absence of genes encoding the respective metabolic pathways. As–Arsenic (+5 arsenate / 3+ arsenite). Grx–Glutaredoxin. P–Phosphate. C4-C– C4-dicarboxylates. G3P - Glycerol-3-P. ISR–Induced systemic response. TxSS–Type 1/2/6 secretion system. WCI–Widespread Colonization Island. Tat–Twin Arginine Translocator. NRPs–Non-ribosomal peptides. Diaz–Diazotrophic. Nitrif–Nitrification.

We have investigated the metabolic capabilities of Mc250 in detail. Our analyses allowed us to infer that Mc250 is highly adapted to the extreme conditions imposed by the environment in which it was isolated, the ferruginous rock fields in the Iron Quadrangle, as well as to its plant host (Fig 8).

Mc250 has a complex network of pathways associated with the degradation of phenolic compounds. These pathways are interrelated in an intricate adaptive network (Fig 3D). Because virtually all pathways culminate in the synthesis of pyruvate, acetyl-coA or succinyl-coA, intermediates of the glycolytic pathway and TCA, we hypothesize that Mc250 can use, as an alternative source of carbon, various phenolic compounds present in the soil or produced by plants, which generate these as a defense against pathogens. This means that Mc250 is well-suited to survive in contact with plants, even in the presence of these toxic compounds. In addition, Mc250 apparently can metabolize distinctive carbohydrates, many from plant origin, using a repertoire of genes associated with the acquisition and metabolism of C4-dicarboxylates (malate, succinate, fumarate, succinate), which can be used as alternative carbon source. These compounds have been reported as present in root exudates [49] and may also be associated with chemotactic events in the process of attraction of bacteria associated with plants [50]. Thus, it is possible that the Iron Quadrangle plants can secrete these compounds as a way to attract not only Mc250 but other potential PGPB as an additional adaptation resource in a soil with highly restrictive characteristics to most plants.

Despite this ability to use these plant metabolites, we found that Mc250 possesses all genes of the propanediol pathway, even though it lacks the biosynthesis pathways of IAA, acetoin and butanediol. Mc250 has all genes necessary for complete nitrogen metabolism, which is evidence that Mc250 possesses the ability to act as a denitrifying organism. Although no ability to solubilize phosphate has been identified due to the absence of important genes in this pathway, Mc250 has the potential to internalize G3P produced by the plant through a specialized ABC transporter, based on results that have been reported for *Escherichia coli* [51]. Although G3P-input-mediated phosphate acquisition may be a secondary mechanism of phosphate acquisition, Mc250 has the high-affinity Pst system (described in *Burkholderia multivorans* [52]), and therefore both systems could together provide the bacterium with phosphate, a fundamental component of cellular metabolism. The existence of two such systems in the genome might be explained by the fact that phosphate concentration in the soils of Brazilian rupestrian fields is extremely low [53, 54].

The presence of two copies of the apparatus coding for the T1SS, four copies of genes encoding RTX-like adhesins, two gene clusters associated with widespread colonization island, and the presence of a complete T2SS coded by Gsp Proteins, may be associated with broad aggregation capacity and biofilm formation [40, 55, 56], which could provide protection for Mc250 against other organisms present in the environment, facilitating plant tissue colonization [57]. At the same time, a repertoire of genes associated with metabolism of metals such as zinc, cadmium, copper, and iron, coupled with the proven ability to remove arsenic from the medium [19], provide strong evidence of the tolerance and ability of Mc250 as a bioremediator. This ability of Mc250 could reduce the bioavailability of these metals to plant tissues, even if these plants can bioaccumulate these compounds [58].

In addition to the biotechnological potential to resist and remove pollutants, we have shown that Mc250 is also capable to inhibit important plant pathogens. The culture supernatant of Mc250 was shown to have a nematicide effect, killing up to 100% of the nematodes after 48 hours of immersion in bacterial supernatant. The nematodes of the genus *Pratylenchus*, also known as nematodes of root lesions, are recognized worldwide as one of the most serious problems in crops of great economic importance, such as soybean, cotton, corn, coffee, and forage [59]. In Brazil, *P*. *brachyurus* causes widespread damages, with significant economic losses in several crops and in various regions of the country [60]. Mc250 was also able to inhibit the growth of *Xanthomonas citri* subsp. *citri* A306 both *in vitro* when co-inoculated with this pathogen in leaves of *Citrus sinensis*. The strain A306 is a causative agent of citrus canker in a wide diversity of citrus hosts, resulting in large losses in the production of fruits and orange juice [61].

Therefore, the ability to tolerate and remove metals [19], to act as a nematicide and bactericide in association with the ability to metabolize phenolic compounds produced by plants suggests that *A*. *faecalis* strain Mc250 can be explored as an important bioinoculant of agricultural interest.

## Supporting information

S1 Fig Pan- and core genome analysis. (A) Pie chart summarizing the numbers of core and acessory genes identified in the pangenome (B) Graph representing the pan-genome (blue) and core-genome (red) of the 37 *Alcaligenes faecalis* analyzed genomes.
(TIF)

S2 Fig Comparative analysis of presence or absence of genes associated with antibiotic and toxin resistance and bacteriocin production (A) and metal resistance (B) in the Mc250 genome with relation to the other 12 strains of the same species investigated.
(TIF)

S3 Fig Comparative analysis of presence or absence of genes associated with stress adaptation (A) and involved with DNA repair (B) in the Mc250 genome with relation to the other 12 strains of the same species investigated.
(TIF)

S4 Fig Comparative analysis of genes associated with sulfur metabolism (A) and biosynthesis of cysteine (B) in the genome of Mc250 with respect to the other four *Af* strains investigated. (C) Integrative analysis of sulfur acquisition and metabolism pathways in association with cysteine synthesis pathways, which once synthesized can act as a precursor of glutathione synthesis (GSH), fundamental to the process of adaptation to oxidative stress. (D) Systems involved with potassium homeostasis in the SfFG3 genome. (E) Secretory systems identified in the genome of SlFG3. TXSS–Type (1, 2 and 3) secretion systems. WCI–Widespread Colonization Island. Tat–Twin arginine translocationg. OM–Outer membrane. IM–Inner membrane.
(TIF)

S1 Table Unique genes of Mc250 identified after pan- and core-genome analysis. (DOCX)

S2 Table Genome islands features of Mc250. (XLSX)

S3 Table Genetic and metabolic information related to the genes described in the text. (DOCX)

S4 Table Identified secondary metabolite clusters in Mc250 genome. (DOCX)
