# Supplementary figures and images for: Complete genome sequence and analysis of Alcaligenes faecalis strain Mc250, a new potential plant bioinoculant
Source: PLoS One. 2020 Nov 5;15(11):e0241546. doi: 10.1371/journal.pone.0241546 (PMC7643998; doi:10.1371/journal.pone.0241546)

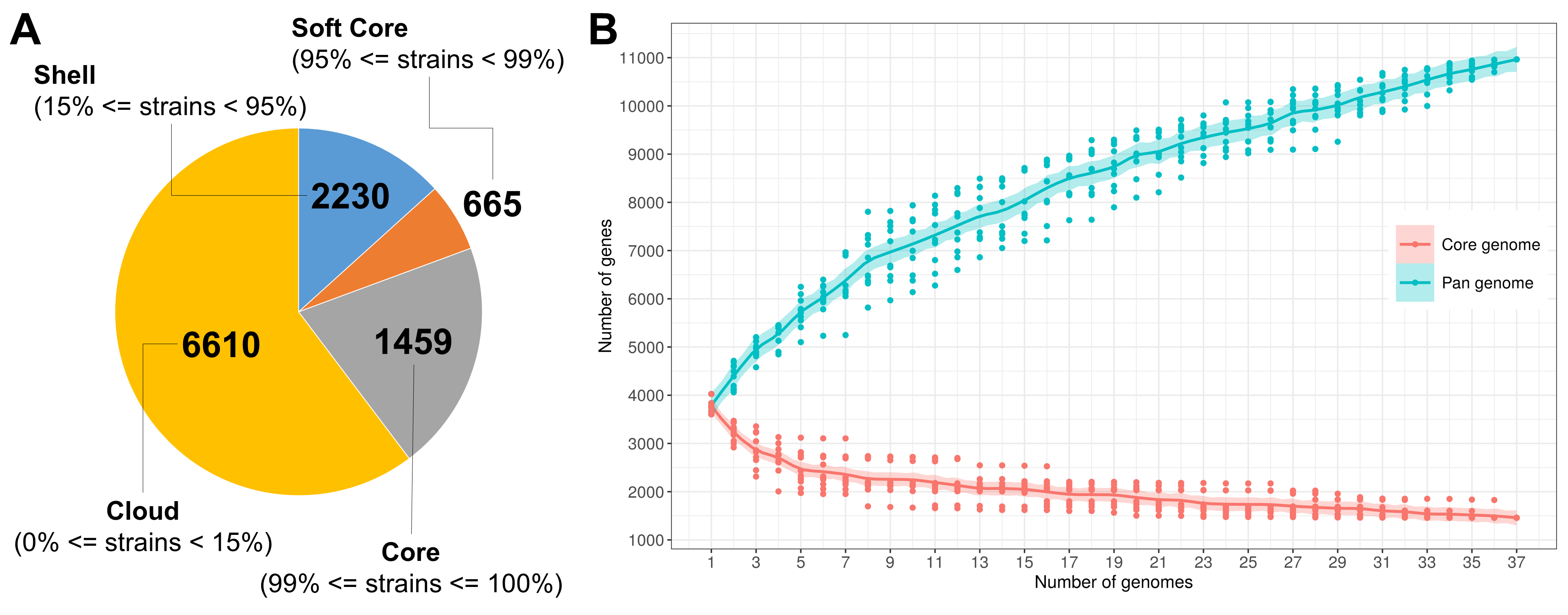

Supplement: S1 Fig — (A) Pie chart summarizing the numbers of core and acessory genes identified in the pangenome (B) Graph representing the pan-genome (blue) and core-genome (red) of the 37 Alcaligenes faecalis analyzed genomes. (TIF) [file pone.0241546.s001.tif]

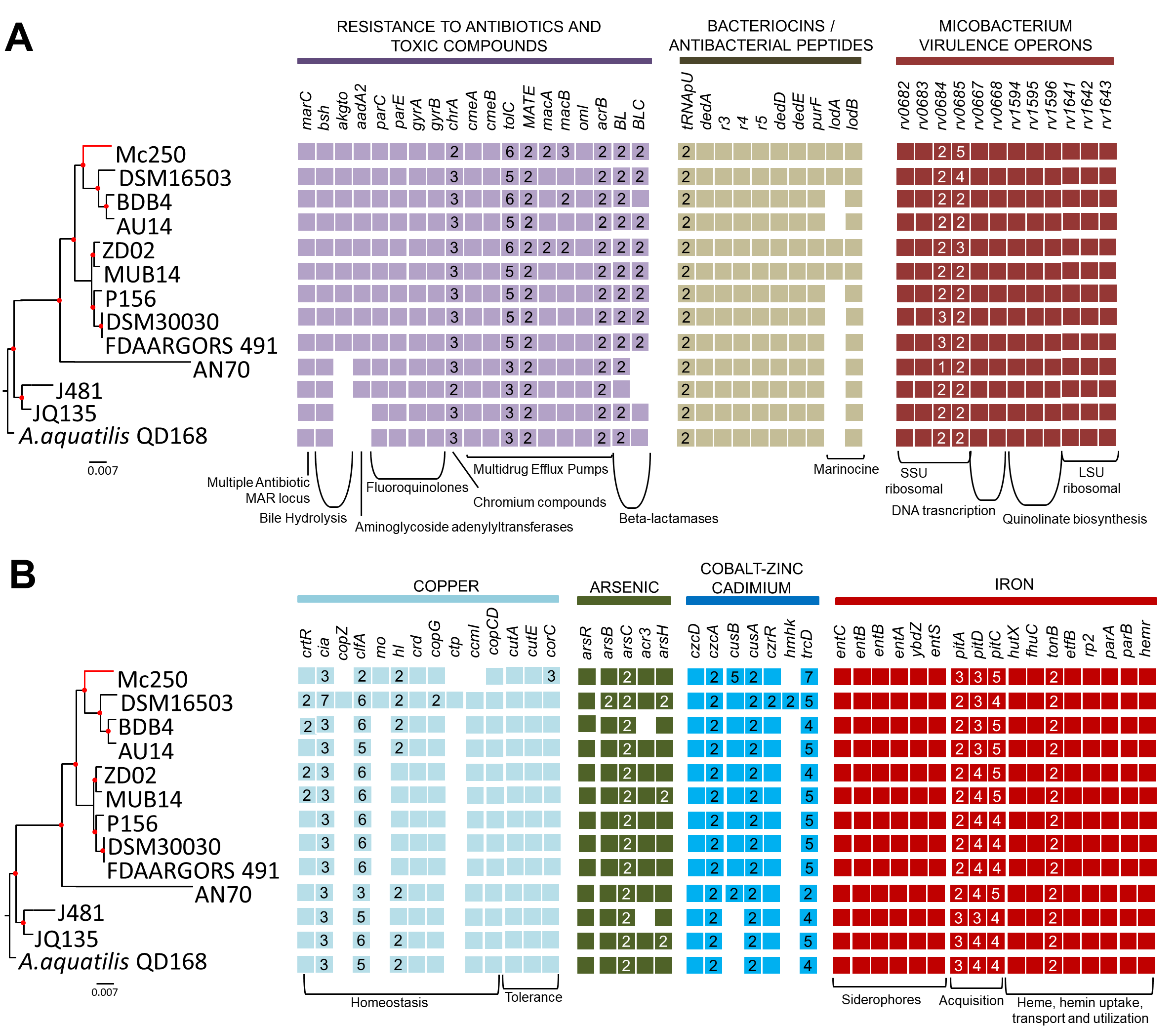

Supplement: S2 Fig — Comparative analysis of presence or absence of genes associated with antibiotic and toxin resistance and bacteriocin production (A) and metal resistance (B) in the Mc250 genome with relation to the other 12 strains of the same species investigated. (TIF) [file pone.0241546.s002.tif]

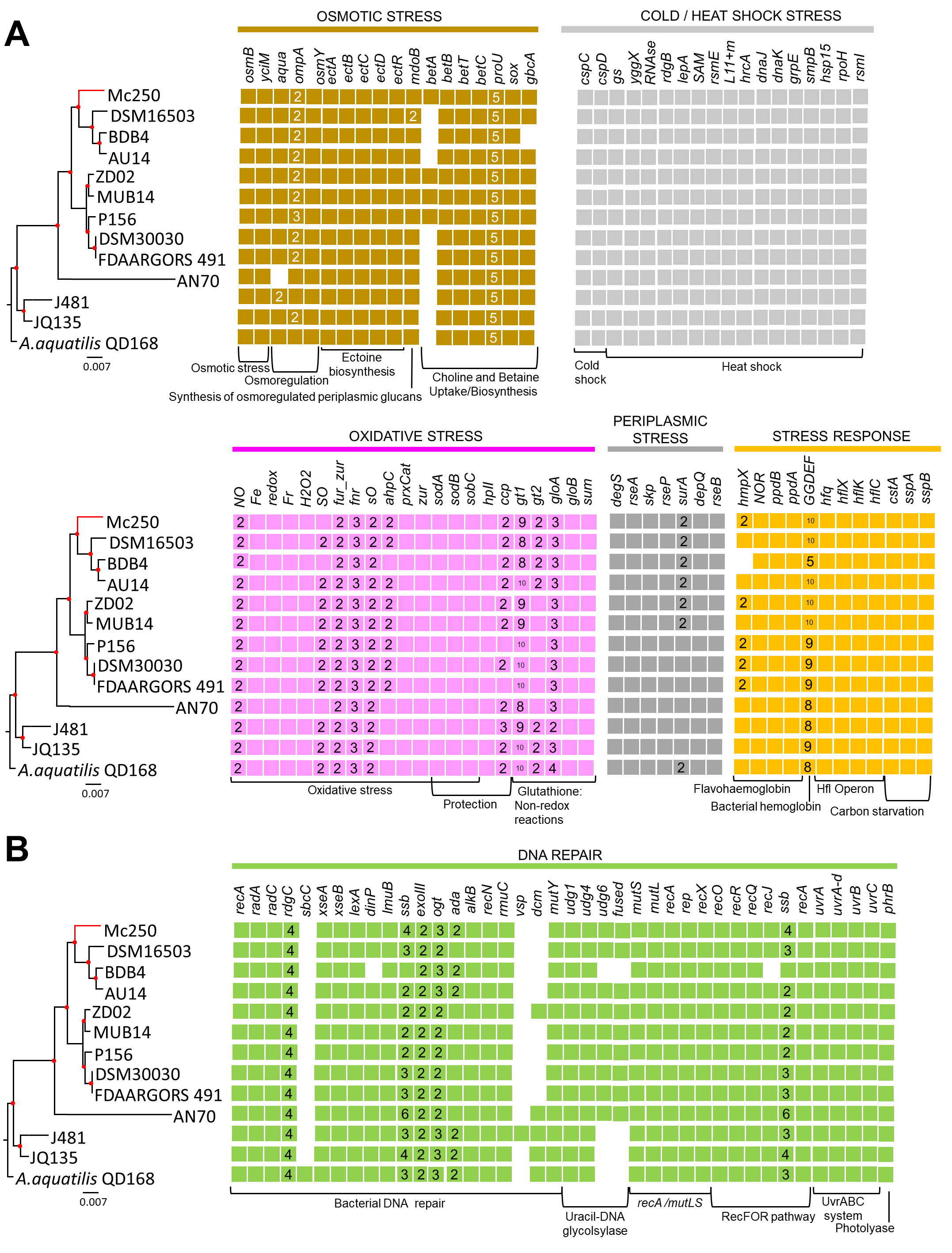

Supplement: S3 Fig — Comparative analysis of presence or absence of genes associated with stress adaptation (A) and involved with DNA repair (B) in the Mc250 genome with relation to the other 12 strains of the same species investigated. (TIF) [file pone.0241546.s003.tif]

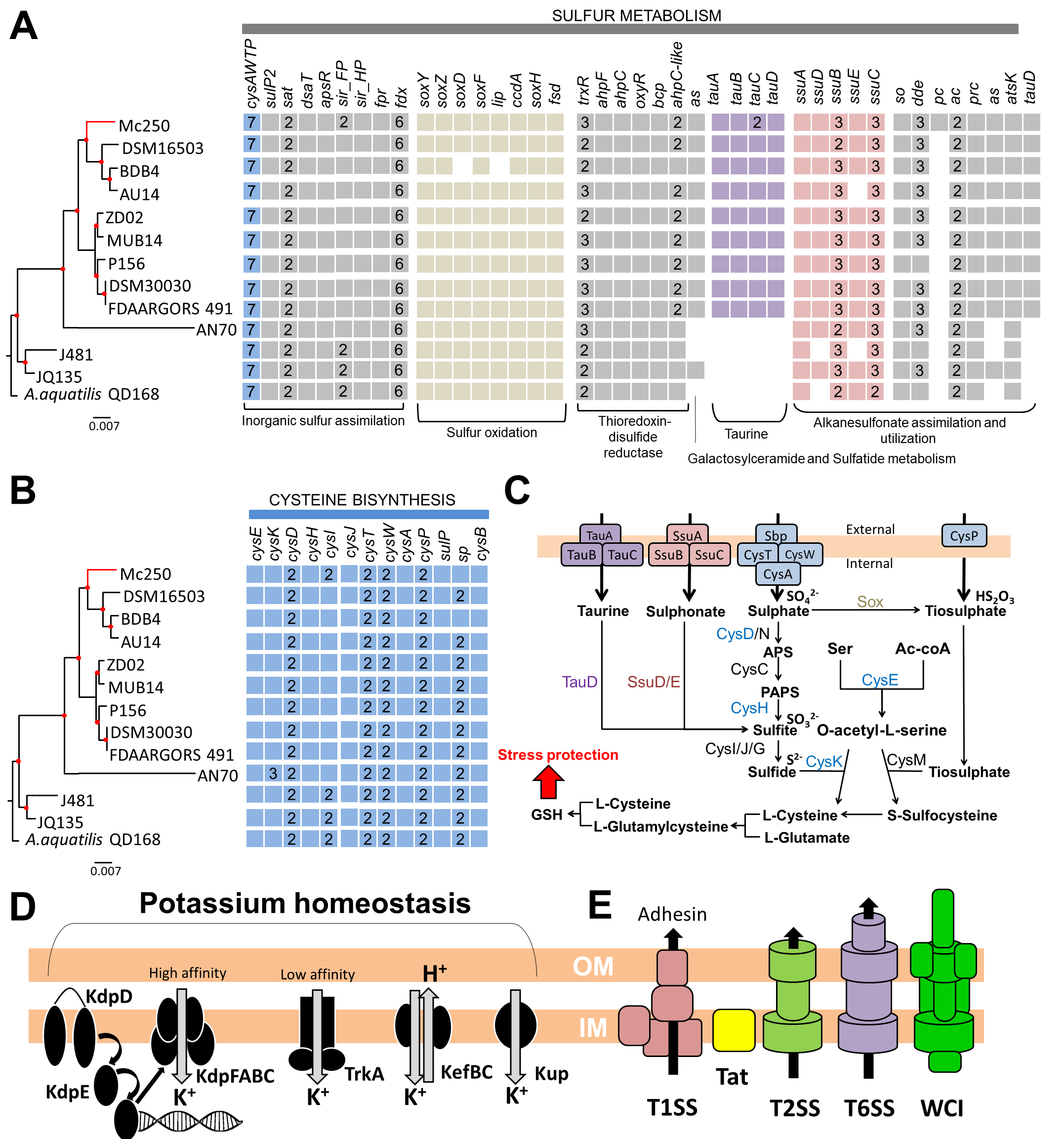

Supplement: S4 Fig — Comparative analysis of genes associated with sulfur metabolism (A) and biosynthesis of cysteine (B) in the genome of Mc250 with respect to the other four Af strains investigated. (C) Integrative analysis of sulfur acquisition and metabolism pathways in association with cysteine synthesis pathways, which once synthesized can act as a precursor of glutathione synthesis (GSH), fundamental to the process of adaptation to oxidative stress. (D) Systems involved with potassium homeostasis in the SfFG3 genome. (E) Secretory systems identified in the genome of SlFG3. TXSS–Type (1, 2 and 3) secretion systems. WCI–Widespread Colonization Island. Tat–Twin arginine translocationg. OM–Outer membrane. IM–Inner membrane. (TIF) [file pone.0241546.s004.tif]
