# Supplementary material for: Complete genome sequence and analysis of Alcaligenes faecalis strain Mc250, a new potential plant bioinoculant
Source: PLoS One. 2020 Nov 5;15(11):e0241546. doi: 10.1371/journal.pone.0241546 (PMC7643998; doi:10.1371/journal.pone.0241546)
Supplement: S1 Table — (DOCX) [file pone.0241546.s005.docx]

**S1 Table** Unique genes of *Af*Mc250

| **Mc250 Locus tag** | **Prodcut** |
| --- | --- |
| DUD43_00080 | potassium transporter |
| DUD43_00220 | autotransporter outer membrane beta-barrel domain-containing protein |
| DUD43_00375 | MFS transporter |
| DUD43_00450 | hypothetical protein |
| DUD43_00695 | DUF2157 domain-containing protein |
| DUD43_00770 | hypothetical protein |
| DUD43_01090 | pseudouridine synthase |
| DUD43_01320 | glycosyl transferase |
| DUD43_01655 | hypothetical protein |
| DUD43_02235 | ATPase |
| DUD43_02245 | hypothetical protein |
| DUD43_02250 | hypothetical protein |
| DUD43_02255 | hypothetical protein |
| DUD43_02260 | MFS transporter |
| DUD43_02320 | hypothetical protein |
| DUD43_02365 | TetR/AcrR family transcriptional regulator |
| DUD43_02525 | TonB-dependent siderophore receptor |
| DUD43_02775 | autotransporter outer membrane beta-barrel domain-containing protein |
| DUD43_02780 | hypothetical protein |
| DUD43_02905 | SDR family NAD%28P%29-dependent oxidoreductase |
| DUD43_02910 | AraC family transcriptional regulator |
| DUD43_02920 | ferrous iron transport protein A |
| DUD43_03005 | helicase |
| DUD43_03010 | DUF1156 domain-containing protein |
| DUD43_03015 | Appr-1-p processing protein |
| DUD43_03020 | DUF4433 domain-containing protein |
| DUD43_03025 | DUF3780 domain-containing protein |
| DUD43_03030 | DUF499 domain-containing protein |
| DUD43_03035 | hypothetical protein |
| DUD43_03040 | integrase |
| DUD43_03080 | DUF4111 domain-containing protein |
| DUD43_03090 | hypothetical protein |
| DUD43_03095 | disulfide bond formation protein B |
| DUD43_03235 | hypothetical protein |
| DUD43_03445 | hypothetical protein |
| DUD43_04200 | oxidoreductase |
| DUD43_04210 | PepSY domain-containing protein |
| DUD43_04430 | hypothetical protein |
| DUD43_04505 | hypothetical protein |
| DUD43_04560 | hypothetical protein |
| DUD43_04565 | MarR family transcriptional regulator |
| DUD43_04650 | hypothetical protein |
| DUD43_04930 | hypothetical protein |
| DUD43_04985 | FAD-dependent monooxygenase |
| DUD43_05090 | hypothetical protein |
| DUD43_05250 | hypothetical protein |
| DUD43_05415 | hypothetical protein |
| DUD43_05760 | glycosyltransferase family 1 protein |
| DUD43_05800 | hypothetical protein |
| DUD43_05870 | hypothetical protein |
| DUD43_05875 | DUF1176 domain-containing protein |
| DUD43_06220 | flagellar hook-length control protein FliK |
| DUD43_06440 | hypothetical protein |
| DUD43_06445 | DUF2591 domain-containing protein |
| DUD43_06450 | hypothetical protein |
| DUD43_06465 | hypothetical protein |
| DUD43_06475 | hypothetical protein |
| DUD43_06490 | hypothetical protein |
| DUD43_06505 | hypothetical protein |
| DUD43_06510 | helix-turn-helix domain-containing protein |
| DUD43_06520 | hypothetical protein |
| DUD43_06555 | hypothetical protein |
| DUD43_06560 | HNH endonuclease |
| DUD43_06565 | TerS protein |
| DUD43_06570 | terminase large subunit |
| DUD43_06575 | phage portal protein |
| DUD43_06580 | HK97 family phage prohead protease |
| DUD43_06585 | phage major capsid protein |
| DUD43_06590 | hypothetical protein |
| DUD43_06595 | phage gp6-like head-tail connector protein |
| DUD43_06610 | DUF3168 domain-containing protein |
| DUD43_06630 | phage tail tape measure protein |
| DUD43_06655 | SGNH/GDSL hydrolase family protein |
| DUD43_06660 | hypothetical protein |
| DUD43_06675 | DUF2514 family protein |
| DUD43_06680 | SOS response-associated peptidase |
| DUD43_06720 | sensor domain-containing diguanylate cyclase |
| DUD43_06920 | hypothetical protein |
| DUD43_07080 | EamA family transporter |
| DUD43_07085 | hypothetical protein |
| DUD43_07180 | site-specific integrase |
| DUD43_07185 | DNA-binding protein |
| DUD43_07190 | hypothetical protein |
| DUD43_07200 | hypothetical protein |
| DUD43_07210 | hypothetical protein |
| DUD43_07225 | DUF1566 domain-containing protein |
| DUD43_07270 | hypothetical protein |
| DUD43_07275 | S24 family peptidase |
| DUD43_07280 | hypothetical protein |
| DUD43_07285 | hypothetical protein |
| DUD43_07290 | MarR family transcriptional regulator |
| DUD43_07310 | hypothetical protein |
| DUD43_07320 | hypothetical protein |
| DUD43_07325 | hypothetical protein |
| DUD43_07330 | terminase small subunit |
| DUD43_07335 | hypothetical protein |
| DUD43_07340 | hypothetical protein |
| DUD43_07345 | hypothetical protein |
| DUD43_07350 | hypothetical protein |
| DUD43_07355 | hypothetical protein |
| DUD43_07360 | DUF4043 family protein |
| DUD43_07365 | hypothetical protein |
| DUD43_07370 | hypothetical protein |
| DUD43_07375 | hypothetical protein |
| DUD43_07380 | hypothetical protein |
| DUD43_07385 | hypothetical protein |
| DUD43_07390 | hypothetical protein |
| DUD43_07395 | hypothetical protein |
| DUD43_07400 | hypothetical protein |
| DUD43_07405 | hypothetical protein |
| DUD43_07410 | hypothetical protein |
| DUD43_07415 | lysozyme |
| DUD43_07425 | hypothetical protein |
| DUD43_07435 | hypothetical protein |
| DUD43_07450 | hypothetical protein |
| DUD43_07520 | hypothetical protein |
| DUD43_07540 | hypothetical protein |
| DUD43_07545 | hypothetical protein |
| DUD43_07815 | ATP-binding protein |
| DUD43_07820 | hypothetical protein |
| DUD43_07905 | hypothetical protein |
| DUD43_07960 | hypothetical protein |
| DUD43_08155 | MFS transporter |
| DUD43_08215 | hypothetical protein |
| DUD43_08240 | DUF2075 domain-containing protein |
| DUD43_08245 | hypothetical protein |
| DUD43_08250 | DUF2514 family protein |
| DUD43_08255 | DUF3320 domain-containing protein |
| DUD43_08390 | hypothetical protein |
| DUD43_08820 | hypothetical protein |
| DUD43_08860 | HNH endonuclease |
| DUD43_08875 | hypothetical protein |
| DUD43_08890 | single-stranded DNA-binding protein |
| DUD43_08910 | hypothetical protein |
| DUD43_08915 | hypothetical protein |
| DUD43_08920 | hypothetical protein |
| DUD43_08935 | hypothetical protein |
| DUD43_08940 | hypothetical protein |
| DUD43_08945 | MarR family transcriptional regulator |
| DUD43_08960 | hypothetical protein |
| DUD43_08965 | hypothetical protein |
| DUD43_08985 | hypothetical protein |
| DUD43_09020 | hypothetical protein |
| DUD43_09025 | hypothetical protein |
| DUD43_09035 | hypothetical protein |
| DUD43_09040 | hypothetical protein |
| DUD43_09055 | nucleoid-associated protein NdpA |
| DUD43_09060 | hypothetical protein |
| DUD43_09065 | hypothetical protein |
| DUD43_09085 | hypothetical protein |
| DUD43_09115 | hypothetical protein |
| DUD43_09150 | hypothetical protein |
| DUD43_09155 | hypothetical protein |
| DUD43_09160 | DUF2514 family protein |
| DUD43_09165 | hypothetical protein |
| DUD43_09170 | hypothetical protein |
| DUD43_09180 | hypothetical protein |
| DUD43_09185 | hypothetical protein |
| DUD43_09190 | hypothetical protein |
| DUD43_09195 | hypothetical protein |
| DUD43_09260 | hypothetical protein |
| DUD43_09340 | hypothetical protein |
| DUD43_09475 | ATP-binding protein |
| DUD43_09480 | hypothetical protein |
| DUD43_09485 | hypothetical protein |
| DUD43_09530 | hypothetical protein |
| DUD43_09730 | hypothetical protein |
| DUD43_09760 | MFS transporter |
| DUD43_09765 | TetR/AcrR family transcriptional regulator |
| DUD43_10040 | MFS transporter |
| DUD43_10225 | prolyl-tRNA synthetase associated domain-containing protein |
| DUD43_10250 | DsbA family protein |
| DUD43_10260 | hypothetical protein |
| DUD43_10295 | hypothetical protein |
| DUD43_10300 | DUF523 domain-containing protein |
| DUD43_10305 | LysR family transcriptional regulator |
| DUD43_10310 | PhzF family phenazine biosynthesis protein |
| DUD43_10315 | RidA family protein |
| DUD43_10335 | ligand-binding protein SH3 |
| DUD43_10765 | LysR family transcriptional regulator |
| DUD43_10850 | hypothetical protein |
| DUD43_10855 | hypothetical protein |
| DUD43_11050 | zinc-binding alcohol dehydrogenase family protein |
| DUD43_11055 | transcriptional regulator |
| DUD43_11165 | hypothetical protein |
| DUD43_11215 | hypothetical protein |
| DUD43_11510 | Lrp/AsnC family transcriptional regulator |
| DUD43_11895 | DMT family transporter |
| DUD43_11945 | hypothetical protein |
| DUD43_11950 | hypothetical protein |
| DUD43_11955 | hypothetical protein |
| DUD43_12390 | hypothetical protein |
| DUD43_12415 | LysR family transcriptional regulator |
| DUD43_12420 | MFS transporter |
| DUD43_12435 | antibiotic biosynthesis monooxygenase |
| DUD43_12440 | NADPH:quinone oxidoreductase |
| DUD43_12445 | ArsR family transcriptional regulator |
| DUD43_12650 | aminotransferase class III-fold pyridoxal phosphate-dependent enzyme |
| DUD43_12655 | helix-turn-helix domain-containing protein |
| DUD43_12965 | hypothetical protein |
| DUD43_13570 | porin |
| DUD43_13645 | FAD:protein FMN transferase |
| DUD43_13660 | DUF1275 domain-containing protein |
| DUD43_13785 | hypothetical protein |
| DUD43_13790 | ABC transporter ATP-binding protein |
| DUD43_13795 | ABC transporter permease |
| DUD43_13800 | TolC family protein |
| DUD43_13805 | HlyD family efflux transporter periplasmic adaptor subunit |
| DUD43_13890 | hypothetical protein |
| DUD43_13910 | hypothetical protein |
| DUD43_14080 | hypothetical protein |
| DUD43_14105 | AraC family transcriptional regulator |
| DUD43_14265 | type 1 fimbrial protein |
| DUD43_14330 | hypothetical protein |
| DUD43_14540 | hypothetical protein |
| DUD43_14545 | hypothetical protein |
| DUD43_14550 | hypothetical protein |
| DUD43_15155 | DUF2834 domain-containing protein |
| DUD43_15160 | N-acetyltransferase |
| DUD43_15165 | GFA family protein |
| DUD43_15170 | hypothetical protein |
| DUD43_15175 | hypothetical protein |
| DUD43_15405 | hypothetical protein |
| DUD43_15515 | autotransporter outer membrane beta-barrel domain-containing protein |
| DUD43_15780 | hypothetical protein |
| DUD43_16195 | hypothetical protein |
| DUD43_16570 | magnesium transporter CorA family protein |
| DUD43_16575 | magnesium transporter CorA family protein |
| DUD43_16580 | magnesium transporter CorA family protein |
| DUD43_16890 | acyltransferase |
| DUD43_16895 | hypothetical protein |
| DUD43_16905 | glycosyltransferase |
| DUD43_16910 | O-antigen ligase domain-containing protein |
| DUD43_16915 | glycosyltransferase |
| DUD43_16955 | hypothetical protein |
| DUD43_16975 | dTDP-4-dehydrorhamnose reductase |
| DUD43_17260 | sulfate ABC transporter substrate-binding protein |
| DUD43_17265 | formate dehydrogenase-N subunit alpha |
| DUD43_17295 | TetR family transcriptional regulator |
| DUD43_17370 | hypothetical protein |
| DUD43_17410 | glycosyltransferase |
| DUD43_17415 | hypothetical protein |
| DUD43_17440 | hypothetical protein |
| DUD43_17500 | hypothetical protein |
| DUD43_17795 | hypothetical protein |
| DUD43_17995 | AraC family transcriptional regulator |
| DUD43_18225 | APH%286%29 family putative aminoglycoside O-phosphotransferase |
| DUD43_18260 | GNAT family N-acetyltransferase |
| DUD43_18990 | hypothetical protein |
| DUD43_19030 | hypothetical protein |
