# Supplementary material for: Complete genome sequence and analysis of Alcaligenes faecalis strain Mc250, a new potential plant bioinoculant
Source: PLoS One. 2020 Nov 5;15(11):e0241546. doi: 10.1371/journal.pone.0241546 (PMC7643998; doi:10.1371/journal.pone.0241546)
Supplement: S3 Table — (DOCX) [file pone.0241546.s007.docx]

**S3 Table** Genetic and metabolic information related to the genes described in the text.

| **Metabolism of Aromatic Compounds: Peripheral pathways** | | | | | |
| --- | --- | --- | --- | --- | --- |
| Pathway | Gene | Product | EC number | Locus Tags |  |
| Salicylate ester degradation | *salA* | Salicylate hydroxylase | 1.14.13.1 | QFY77992.1 |  |
|  |  |  |  | QFY76725.1 |  |
|  |  |  |  | QFY79911.1 |  |
|  |  |  |  | QFY77761.1 |  |
|  | *salE* | Salicylate esterase |  | QFY77670.1 |  |
| Phenol hydroxylase | *dmpK* | Phenol hydroxylase, assembly protein |  | QFY77860.1 |  |
|  | *dmpM* | Phenol hydroxylase, P2 regulatory component | 1.14.13.7 | QFY77862.1 |  |
|  | *dmpL* | Phenol hydroxylase, P1 regulatory component | 1.14.13.7 | QFY77861.1 |  |
|  | *dmpN* | Phenol hydroxylase, P3 regulatory component | 1.14.13.7 | QFY77863.1 |  |
|  | *dmpO* | Phenol hydroxylase, P4 regulatory component | 1.14.13.7 | QFY77864.1 |  |
|  | *dmpP* | Phenol hydroxylase, FAD- and [2Fe-2S]-containing reductase component |  | QFY77865.1 |  |
| Toluene degradation | *tad* | Toluenesulfonate zinc-independent alcohol dehydrogenase |  | QFY76851.1 |  |
| Biphenyl Degradation | *bphH* | 2-keto-4-pentenoate hydratase | 4.2.1.80 | QFY78761.1 |  |
|  |  |  |  | QFY76497.1 |  |
|  | *bphC* | Biphenyl-2,3-diol 1,2-dioxygenase | 1.13.11.39 | QFY77991.1 |  |
|  |  | Biphenyl-2,3-diol 1,2-dioxygenase III-related protei |  | QFY78555.1 |  |
|  | *bphD* | 2-hydroxy-6-oxo-6-phenylhexa-2,4-dienoate hydrolase | 3.7.1.- | QFY78760.1 |  |
|  | *bphI* | 4-hydroxy-2-oxovalerate aldolase | 4.1.3.39 | QFY78763.1 |  |
|  | *bphJ* | Acetaldehyde dehydrogenase, acetylating, in gene cluster for degradation of phenols, cresols, catechol | 1.2.1.10 | QFY78762.1 |  |
| Benzoate degradation | *benB* | Benzoate 1,2-dioxygenase beta subunit | 1.14.12.10 | QFY77868.1 |  |
|  | *benA* | Benzoate 1,2-dioxygenase alpha subunit | 1.14.12.10 | QFY77867.1 |  |
|  | *benC* | Benzoate dioxygenase, ferredoxin reductase component |  | QFY77869.1 |  |
|  | *benD* | 1,2-dihydroxycyclohexa-3,5-diene-1-carboxylate dehydrogenase | 1.3.1.25 | QFY77647.1 |  |
|  |  |  |  | QFY77870.1 |  |
|  | *benK* | Benzoate MFS transporter |  | QFY78437.1 |  |
|  |  |  |  | QFY77871.1 |  |
|  | *benE2* | Benzoate transport protein |  | QFY78309.1 |  |
|  |  |  |  | QFY77034.1 |  |
|  |  |  |  | QFY77748.1 |  |
|  | *benR* | benABC operon transcriptional activator |  | QFY77239.1 |  |
|  | *ohbA* | Ortho-halobenzoate 1,2-dioxygenase beta-ISP protein |  | QFY78997.1 |  |
|  | *ohbB* | Ortho-halobenzoate 1,2-dioxygenase alpha-ISP protein |  | QFY78996.1 |  |
| p-Hydroxybenzoate degradation | *ht* | 4-hydroxybenzoate transporter |  | QFY78764.1 |  |
| Chloroaromatic degradation | *catD* | Beta-ketoadipate enol-lactone hydrolase | 3.1.1.24 | QFY77746.1 |  |
|  | *catF* | Acetyl-CoA acetyltransferase | 2.3.1.9 | QFY77585.1 |  |
|  | *catI* | 3-oxoadipate CoA-transferase subunit B | 2.8.3.6 | QFY79776.1 |  |
|  | *catJ* | 3-oxoadipate CoA-transferase subunit A |  | QFY77745.1 |  |
| Catechol branch of b-ketoadipate pathway | *catA* | Catechol 1,2-dioxygenase | 1.13.11.1 | QFY77744.1 |  |
|  | *catB* | Muconate cycloisomerase | 5.5.1.1 | QFY76634.1 |  |
|  |  |  |  | QFY77743.1 |  |
|  | *catC* | Muconolactone isomerase | 5.3.3.4 | QFY77747.1 |  |
|  | *catD* | Beta-ketoadipate enol-lactone hydrolase | 3.1.1.24 | QFY77746.1 |  |
|  | *catEA* | Succinyl-CoA:3-ketoacid-coenzyme A transferase subunit A | 2.8.3.5 | QFY78559.1 |  |
|  |  | 3-oxoadipate CoA-transferase subunit A | 2.8.3.6 | QFY77745.1 |  |
|  | *catEB* | Succinyl-CoA:3-ketoacid-coenzyme A transferase subunit B | 2.8.3.5 | QFY78558.1 |  |
|  |  | 3-oxoadipate CoA-transferase subunit B | 2.8.3.6 | QFY79776.1 |  |
| Salicylate and gentisate catabolism | *salA* | Salicylate hydroxylase | 1.14.13.1 | QFY77992.1 |  |
|  |  |  |  | QFY76725.1 |  |
|  |  |  |  | QFY79911.1 |  |
|  |  |  |  | QFY77761.1 |  |
|  | *salE* | Salicylate esterase |  | QFY77670.1 |  |
|  | *salD* | 4-hydroxybenzoate transporter |  | QFY78764.1 |  |
|  | *mctI* | Maleylacetoacetate isomerase | 5.2.1.2 | QFY78526.1 |  |
|  |  | Maleate cis-trans isomerase | 5.2.1.1 | QFY79246.1 |  |
|  | *faa* | Fumarylacetoacetase | 3.7.1.2 | QFY76722.1 |  |
|  | *fhfF* | Fumarylacetoacetate hydrolase family protein |  | QFY76240.1 |  |
|  |  |  |  | QFY79537.1 |  |
|  |  |  |  | QFY79798.1 |  |
|  |  |  |  | QFY78217.1 |  |
| N-heterocyclic aromatic compound degradation | *iqoA* | Isoquinoline 1-oxidoreductase alpha subunit | 1.3.99.16 | QFY78968.1 |  |
|  | *iqoB* | Isoquinoline 1-oxidoreductase beta subunit |  | QFY78967.1 |  |
| Homogentisate pathway of aromatic compound degradation | *hd* | Homogentisate 1,2-dioxygenase | 1.13.11.5 | QFY76723.1 |  |
|  | *hppD* | 4-hydroxyphenylpyruvate dioxygenase | 1.13.11.27 | QFY78843.1 |  |
|  | *mai* | Maleylacetoacetate isomerase | 5.2.1.2 | QFY78526.1 |  |
|  | *faa* | Fumarylacetoacetase | 3.7.1.2 | QFY76722.1 |  |
|  | *aaa* | Aromatic-amino-acid aminotransferase | 2.6.1.57 | QFY79512.1 |  |
|  |  |  |  | QFY79489.1 |  |
|  |  |  |  | QFY77640.1 |  |
|  | *hmgR* | Transcriptional regulator, IclR family |  | QFY78751.1 |  |
|  |  |  |  | QFY77394.1 |  |
|  |  |  |  | QFY77110.1 |  |
|  |  |  |  | QFY77059.1 |  |
|  |  |  |  | QFY78852.1 |  |
|  |  |  |  | QFY79031.1 |  |
|  |  |  |  | QFY78948.1 |  |
|  |  |  |  | QFY76232.1 |  |
| Aromatic Aminoacids Catabolism | *ppaZ* | Aldehyde dehydrogenase | 1.2.1.3 | QFY78393.1 |  |
|  | *nacB* | Nitrilotriacetate monooxygenase component B | 1.14.13.- | QFY76257.1 |  |
|  |  |  |  | QFY76260.1 |  |
|  |  |  |  | QFY78361.1 |  |
|  |  |  |  | QFY78672.1 |  |
|  |  |  |  | QFY76499.1 |  |
|  |  |  |  | QFY76863.1 |  |
|  |  |  |  | QFY77674.1 |  |
|  |  | 4-hydroxyphenylacetate 3-monooxygenase, reductase component | 1.6.8.- | QFY79556.1 |  |
| Gentisate degradation | *gd* | Gentisate 1,2-dioxygenase | 1.13.11.4 | QFY76239.1 |  |
|  | *fahF* | Fumarylacetoacetate hydrolase family protein |  | QFY76240.1 |  |
|  |  |  |  | QFY79537.1 |  |
|  |  |  |  | QFY79798.1 |  |
|  |  |  |  | QFY78217.1 |  |
|  | *mai* | Maleylacetoacetate isomerase | 5.2.1.2 | QFY78526.1 |  |
|  | *ht* | 4-hydroxybenzoate transporter |  | QFY78764.1 |  |
| **Nitrogen Metabolism** | | | | | |
| Dissimilatory nitrite reductase | nirS | Cytochrome cd1 nitrite reductase | 1.7.2.1 | QFY78276.1 |  |
|  | nirF | Heme d1 biosynthesis protein NirF |  | QFY78269.1 |  |
|  | nirD | Heme d1 biosynthesis protein NirD |  | QFY78270.1 |  |
|  | nirL | Heme d1 biosynthesis protein NirL |  | QFY78270.1 |  |
|  | nirG | Heme d1 biosynthesis protein NirG |  | QFY78271.1 |  |
|  | nirH | Heme d1 biosynthesis protein NirH |  | QFY78272.1 |  |
|  | nirJ | Heme d1 biosynthesis protein NirJ |  | QFY78273.1 |  |
|  | nirE | Uroporphyrinogen-III methyltransferase | 2.1.1.107 | QFY78267.1 |  |
|  |  |  |  | QFY78274.1 |  |
|  |  |  |  | QFY79593.1 |  |
| Nitrosative stress | *qNOR* | Nitric-oxide reductase, quinol-dependent | 1.7.99.7 | QFY79146.1 |  |
|  | *norR* | Anaerobic nitric oxide reductase transcription regulator |  | QFY79147.1 |  |
|  | *nnrS* | NnrS protein involved in response to NO |  | QFY77759.1 |  |
|  | *nsrR* | Nitrite-sensitive transcriptional repressor |  | QFY76777.1 |  |
| Nitrilase | *nit* | Plant-induced nitrilase, hydrolyses beta-cyano-L-alanine | 3.5.5.1 | QFY79311.1 |  |
|  | *reg* | Transcriptional regulator in custer with plant-induced nitrilase |  | QFY79309.1 |  |
| Ammonia assimilation | *gs* | Glutamine synthetase type I | 6.3.1.2 | QFY78000.1 |  |
|  | *glnE* | Glutamine synthetase adenylyl-L-tyrosine phosphorylase | 2.7.7.89 | QFY78372.1 |  |
|  | *glnD* | [Protein-PII] uridylyltransferase | 2.7.7.59 | QFY78096.1 |  |
|  | *gogDP1* | Glutamate synthase [NADPH] small chain | 1.4.1.13 | QFY76525.1 |  |
|  |  |  |  | QFY79689.1 |  |
|  |  | Glutamate synthase [NADPH] large chain |  | QFY76657.1 |  |
|  | *gogATF* | Ferredoxin-dependent glutamate synthase | 1.4.7.1 | QFY77450.1 |  |
|  | *NRI* | Nitrogen regulation protein NR(I) |  | QFY78108.1 |  |
|  |  |  |  | QFY78192.1 |  |
|  |  |  |  | QFY79073.1 |  |
|  | *PII* | Nitrogen regulatory protein P-II |  | QFY78150.1 |  |
|  |  |  |  | QFY78479.1 |  |
|  | *amt* | Ammonium transporter |  | QFY78480.1 |  |
| Denitrification | *nirV* | Nitrite reductase accessory protein |  | QFY79149.1 |  |
|  | *nirK* | Copper-containing nitrite reductase | 1.7.2.1 | QFY78812.1 |  |
|  |  |  |  | QFY76689.1 |  |
|  |  |  |  | QFY79145.1 |  |
|  | *nirS* | Cytochrome cd1 nitrite reductase | 1.7.2.1 | QFY78276.1 |  |
|  | *qNor* | Nitric-oxide reductase, quinol-dependent | 1.7.99.7 | QFY79146.1 |  |
|  | *nosX* | Nitrous oxide reductase maturation periplasmic protein NosX |  | QFY79337.1 |  |
|  | *nosL* | Nitrous oxide reductase maturation protein, outer-membrane lipoprotein |  | QFY79336.1 |  |
|  | *nosY* | Nitrous oxide reductase maturation transmembrane protein NosY |  | QFY79335.1 |  |
|  | *nosD* | Nitrous oxide reductase maturation protein NosD |  | QFY79888.1 |  |
|  | *nosZ* | Nitrous-oxide reductase | 1.7.99.6 | QFY79333.1 |  |
|  | *nosR* | Nitrous oxide reductase maturation protein NosR |  | QFY79332.1 |  |
|  | *nnrS* | NnrS protein involved in response to NO |  | QFY77759.1 |  |
|  | *dnr* | Nitric oxide -responding transcriptional regulator Dnr (Crp/Fnr family) |  | QFY79317.1 |  |
| **Phosphorous metabolism** | | | | | |
| High affinity P transporter PHO regulon, phosphate and polyphosphate metabolism | *phoU* | Phosphate transport system regulatory protein PhoU |  | QFY77215.1 |  |
|  | *pstS* | Phosphate ABC transporter, periplasmic phosphate-binding protein PstS | 3.A.1.7.1 | QFY76997.1 |  |
|  | *pstA* | Phosphate transport system permease protein PstA |  | QFY76999.1 |  |
|  | *pstB* | Phosphate transport ATP-binding protein PstB |  | QFY77000.1 |  |
|  | *pstC* | Phosphate transport system permease protein PstC |  | QFY76998.1 |  |
|  | *phoR* | Phosphate regulon sensor protein PhoR (SphS) | 2.7.13.3 | QFY78463.1 |  |
|  |  |  |  | QFY79189.1 |  |
|  | *phoB* | Phosphate regulon transcriptional regulatory protein PhoB (SphR) |  | QFY79188.1 |  |
|  | *ppiK* | Polyphosphate kinase | 2.7.4.1 | QFY76996.1 |  |
|  | *ip* | Inorganic pyrophosphatase | 3.6.1.1 | QFY78218.1 |  |
|  | *thb* | NAD(P) transhydrogenase subunit beta subunit | 1.6.1.2 | QFY77083.1 |  |
|  | *tha* | NAD(P) transhydrogenase subunit alpha subunit |  | QFY77082.1 |  |
|  |  |  |  | QFY77081.1 |  |
|  | *phoH* | Phosphate starvation-inducible protein PhoH, predicted ATPase |  | QFY76815.1 |  |
|  |  |  |  | QFY77194.1 |  |
|  | *ppoUh* | Sodium-dependent phosphate transporter |  | QFY77175.1 |  |
|  |  |  |  | QFY77244.1 |  |
|  |  | Phosphate transport system regulatory protein PhoU |  | QFY77215.1 |  |
|  | *ppx* | Exopolyphosphatase | 3.6.1.11 | QFY76995.1 |  |
|  | *ppk* | Polyphosphate kinase | 2.7.4.1 | QFY76996.1 |  |
| **Plant-Prokaryote DOE project** | | | | | |
| Niacin-choline Transport | *cdh* | Choline dehydrogenase | 1.1.99.1 | QFY78722.1 |  |
|  | *badH* | Betaine aldehyde dehydrogenase | 1.2.1.8 | QFY79524.1 |  |
|  | *betT* | High-affinity choline uptake protein BetT |  | QFY78802.1 |  |
|  | *sox* | Sarcosine oxidase beta subunit | 1.5.3.1 | QFY77948.1 |  |
|  | *niaP* | Niacin transporter NiaP |  | QFY78718.1 |  |
|  | *gbcA* | GbcA Glycine betaine demethylase subunit A |  | QFY76875.1 |  |
|  | *nam* | Nicotinamidase | 3.5.1.19 | QFY76658.1 |  |
| **Secondary Metabolism** | | | | | |
| Phenylpropionate Degradation | *ppdB* | 3-phenylpropionate dioxygenase, beta subunit | 1.14.12.19 | QFY78756.1 |  |
|  | *ppdA* | 3-phenylpropionate dioxygenase, alpha subunit |  | QFY78755. |  |
|  | *dcdCD* | 1,2-dihydroxycyclohexa-3,5-diene-1-carboxylate dehydrogenase | 1.3.1.25 | QFY77647.1 |  |
|  |  |  |  | QFY77870.1 |  |
|  | *ppd* | 3-phenylpropionate dioxygenase ferredoxin subunit |  | QFY79851.1 |  |
|  | *ppdH* | 2,3-dihydroxy-2,3-dihydro-phenylpropionate dehydrogenase | 1.3.1.- | QFY78757.1 |  |
| Auxin biosynthesis | *aprT* | Anthranilate phosphoribosyltransferase | 2.4.2.18 | QFY76437.1 |  |
|  | *praI* | Phosphoribosylanthranilate isomerase | 5.3.1.24 | QFY78179.1 |  |
|  | *tsa* | Tryptophan synthase alpha chain | 4.2.1.20 | QFY78602.1 |  |
|  | *tsb* | Tryptophan synthase beta chain |  | QFY78603.1 |  |
| **Sulfur metabolism** | | | | | |
| Inorganic Sulfur Assimilation | *cysAWTP* | Sulfate and thiosulfate import ATP-binding protein CysA | 3.6.3.25 | QFY78259.1 |  |
|  |  | Sulfate transport system permease protein CysW |  | QFY78260.1 |  |
|  |  |  |  | QFY79412.1 |  |
|  |  | Sulfate transport system permease protein CysT |  | QFY78261.1 |  |
|  |  |  |  | QFY79413.1 |  |
|  |  | Sulfate and thiosulfate binding protein CysP |  | QFY78262.1 |  |
|  |  |  |  | QFY79414.1 |  |
|  | *sulP1* | Sulfate permease, Trk-type |  | QFY77626.1 |  |
|  | *sat* | Sulfate adenylyltransferase subunit 1 | 2.7.7.4 | QFY78920.1 |  |
|  |  | Sulfate adenylyltransferase subunit 2 |  | QFY78919.1 |  |
|  | *dsaT* | Sulfate adenylyltransferase, dissimilatory-type |  | QFY78460.1 |  |
|  | *apsR* | Phosphoadenylyl-sulfate reductase [thioredoxin] | 1.8.4.8 | QFY78918.1 |  |
|  | *sirFP* | Sulfite reductase [NADPH] flavoprotein alpha-component | 1.8.1.2 | QFY79591.1 |  |
|  |  |  |  | QFY76949.1 |  |
|  | *sirHP* | Sulfite reductase [NADPH] hemoprotein beta-component |  | QFY79592.1 |  |
|  | *frp* | Ferredoxin--NADP(+) reductase |  | QFY78007.1 |  |
|  | *fdx* | 4Fe-4S ferredoxin, iron-sulfur binding |  | QFY78428.1 |  |
|  |  |  |  | QFY78607.1 |  |
|  |  |  |  | QFY79520.1 |  |
|  |  |  |  | QFY79518.1 |  |
|  |  |  |  | QFY78953.1 |  |
|  |  |  |  | QFY79779.1 |  |
| Sulfur oxidation | *soxY* | Sulfur oxidaton protein SoxY |  | QFY77423.1 |  |
|  | *soxZ* | Sulfur oxidation protein SoxZ |  | QFY77422.1 |  |
|  | *soxD* | Sulfite dehydrogenase cytochrome subunit SoxD |  | QFY78031.1 |  |
|  | *soxF* | Sulfide dehydrogenase [flavocytochrome C] flavoprotein chain precursor | 1.8.2.- | QFY79749.1 |  |
|  | *lip* | Lipocalin-related protein and Bos/Can/Equ allergen |  | QFY77420.1 |  |
|  | *ccdA* | Cytochrome c-type biogenesis protein CcdA (DsbD analog) |  | QFY76765.1 |  |
|  | *soxH* | SoxH protein, homolog |  | QFY76320.1 |  |
|  | *fsd* | cytochrome c subunit of flavocytochrome c sulfide dehydrogenase |  | QFY77421.1 |  |
| Thioredoxin-disulfide reductase | *trx* | Thioredoxin reductase | 1.8.1.9 | QFY78451.1 |  |
|  |  |  |  | QFY78667.1 |  |
|  |  |  |  | QFY78683.1 |  |
|  | *ahpF* | Alkyl hydroperoxide reductase protein F | 1.6.4.- | QFY78914.1 |  |
|  | *ahpC* | Alkyl hydroperoxide reductase protein C | 1.6.4.- | QFY78915.1 |  |
|  | *oxyR* | Hydrogen peroxide-inducible genes activator |  | QFY78010.1 |  |
|  | *bcp* | Thiol peroxidase, Bcp-type | 1.11.1.15 | QFY77195.1 |  |
|  | *ahpC-like* | Alkyl hydroperoxide reductase subunit C-like protein |  | QFY78403.1 |  |
|  |  |  |  | QFY76617.1 |  |
| Galactosylceramide and Sulfatide metabolism | *as* | Arylsulfatase | 3.1.6.1 | QFY79740.1 |  |
| Taurine Utilization | *tauA* | Taurine-binding periplasmic protein TauA |  | QFY77388.1 |  |
|  | *tauB* | Taurine transport ATP-binding protein TauB |  | QFY77389.1 |  |
|  | *tauC* | Taurine transport system permease protein TauC |  | QFY77390.1 |  |
|  |  |  |  | QFY76611. |  |
|  | *tauD* | Alpha-ketoglutarate-dependent taurine dioxygenase | 1.14.11.17 | QFY78477.1 |  |
| Alkanesulfonate assimilation and utilization | *ssuA* | Alkanesulfonates-binding protein |  | QFY76467.1 |  |
|  | *ssuD* | Alkanesulfonate monooxygenase | 1.14.14.5 | QFY76288.1 |  |
|  | *ssuB* | Alkanesulfonates ABC transporter ATP-binding protein |  | QFY79315.1 |  |
|  |  |  |  | QFY79249.1 |  |
|  |  |  |  | QFY79595.1 |  |
|  | *ssuE* | FMN reductase | 1.5.1.29 | QFY76289.1 |  |
|  | *ssuC* | Alkanesulfonates transport system permease protein |  | QFY79721.1 |  |
|  |  |  |  | QFY79882.1 |  |
|  |  |  |  | QFY79908.1 |  |
|  | *so* | Coenzyme F420-dependent N5,N10-methylene tetrahydromethanopterin reductase and related flavin-dependent oxidoreductases  sulfonate monooxygenase |  | QFY79557.1 |  |
|  | *dde* | Probable dibenzothiophene desulfurization enzyme |  | QFY76281.1 |  |
|  |  |  |  | QFY76282.1 |  |
|  |  |  |  | QFY76286.1 |  |
|  | *pc* | ABC-type nitrate/sulfonate/bicarbonate transport systems, periplasmic components |  | QFY76612.1 |  |
|  | *ac* | ABC-type nitrate/sulfonate/bicarbonate transport system, ATPase component |  | QFY76466.1 |  |
|  |  |  |  | QFY76980.1 |  |
|  | *prc* | ABC-type nitrate/sulfonate/bicarbonate transport system, permease component |  | QFY79687.1 |  |
|  | *as* | Arylsulfatase | 3.1.6.1 | QFY79740.1 |  |
|  | *atsK* | Putative alkylsulfatase | 1.14.11.17 | QFY76204.1 |  |
|  | *tauD* | Alpha-ketoglutarate-dependent taurine dioxygenase | 1.14.11.17 | QFY78477.1 |  |
| Amino Acids and Derivatives |  |  |  |  |  |
| Cysteine Biosynthesis | *cysE* | Serine acetyltransferase | 2.3.1.30 | QFY78573.1 |  |
|  | *cysK* | Cysteine synthase B | 2.5.1.47 | QFY78409.1 |  |
|  | *cysD* | Sulfate adenylyltransferase subunit 1 | 2.7.7.4 | QFY78920.1 |  |
|  |  |  |  | QFY78919.1 |  |
|  | *cysH* | Phosphoadenylyl-sulfate reductase [thioredoxin] | 1.8.4.8 | QFY78918.1 |  |
|  | *cysI* | Sulfite reductase [NADPH] flavoprotein alpha-component | 1.8.1.2 | QFY79591.1 |  |
|  | *cysJ* | Sulfite reductase [NADPH] hemoprotein beta-component |  | QFY79592.1 |  |
|  | *cysT* | Sulfate transport system permease protein CysT |  | QFY78261.1 |  |
|  |  |  |  | QFY79413.1 |  |
|  | *cysW* | Sulfate transport system permease protein CysW |  | QFY78260.1 |  |
|  |  |  |  | QFY79412.1 |  |
|  | *cysA* | Sulfate and thiosulfate import ATP-binding protein CysA | 3.6.3.25 | QFY78259.1 |  |
|  | *cysP* | Sulfate and thiosulfate binding protein CysP |  | QFY78262.1 |  |
|  |  |  |  | QFY79414.1 |  |
|  | *sulP* | Sulfate permease, Trk-type |  | QFY77626.1 |  |
|  | *sp* | Sulfate permease |  | pseudogene |  |
|  | *cysB* | Cys regulon transcriptional activator CysB |  | QFY77016.1 |  |
| **Resistance to antibiotics and toxic compounds** | | | | | |
| Multiple Antibiotic Resistance MAR locus | *marC* | Stress protection protein MarC |  | QFY77436.1 |  |
| Bile hydrolysis | *Bsh* | Choloylglycine hydrolase | 3.5.1.24 | QFY78429.1 |  |
|  | *akgto* | Alpha-ketoglutarate-dependent taurine dioxygenase | 1.14.11.17 | QFY78477.1 |  |
| Aminoglycoside adenylyltransferases | *aadA2* | Spectinomycin 9-O-adenylyltransferase |  | QFY76751.1 |  |
| Resistance to fluoroquinolones | *parC* | Topoisomerase IV subunit A | 5.99.1.- | QFY78356.1 |  |
|  | *parE* | Topoisomerase IV subunit B | 5.99.1.- | QFY78357.1 |  |
|  | *gyrA* | DNA gyrase subunit A | 5.99.1.3 | QFY78422.1 |  |
|  | *gyrB* | DNA gyrase subunit B | 5.99.1.3 | QFY76184.1 |  |
| Resistance to chromium compounds | *chrA* | Chromate transport protein ChrA |  | QFY76600.1 |  |
|  |  |  |  | QFY79385.1 |  |
| Multidrug Resistance Efflux Pumps | *cmeA* | RND efflux system, membrane fusion protein CmeA |  | QFY78120.1 |  |
|  | *cmeB* | RND efflux system, inner membrane transporter CmeB |  | QFY78119.1 |  |
|  | *tolC* | RND efflux system, outer membrane lipoprotein CmeC |  | QFY78118.1 |  |
|  |  |  |  | QFY78202.1 |  |
|  |  |  |  | QFY78725.1 |  |
|  |  |  |  | QFY79724.1 |  |
|  |  |  |  | QFY79181.1 |  |
|  |  |  |  | QFY78788.1 |  |
|  | *MATE* | Multidrug and toxin extrusion (MATE) family efflux pump YdhE/NorM, homolog |  | QFY78535.1 |  |
|  |  |  |  | QFY78905.1 |  |
|  | *macA* | Macrolide-specific efflux protein MacA |  | QFY78204.1 |  |
|  |  |  |  | QFY77039.1 |  |
|  | *macB* | Macrolide export ATP-binding/permease protein MacB | 3.6.3.- | QFY78203.1 |  |
|  |  |  |  | QFY78686.1 |  |
|  |  |  |  | QFY77040.1 |  |
|  | *oml* | RND efflux system, outer membrane lipoprotein, NodT family |  | QFY79154.1 |  |
|  | *acrB* | RND multidrug efflux transporter; Acriflavin resistance protein |  | QFY78805.1 |  |
|  |  |  |  | QFY79010.1 |  |
| Beta-lactamase | *BL* | Beta-lactamase | 3.5.2.6 | QFY78370.1 |  |
|  |  |  |  | QFY79440.1 |  |
|  | *BLC* | Beta-lactamase class C and other penicillin binding proteins | 3.5.2.6 | QFY78099.1 |  |
|  |  |  |  | QFY78461.1 |  |
| **Bacteriocins/Antibacterial Peptides** | | | | | |
| Colicin V and Bacteriocin Production Cluster | *tRNApU* | tRNA pseudoridine synthase A | 4.2.1.70 | QFY78146.1 |  |
|  |  |  |  | QFY78182.1 |  |
|  | *dedA* | DedA protein |  | QFY76947.1 |  |
|  | *r3* | Acetyl-coenzyme A carboxyl transferase beta chain | 6.4.1.2 | QFY78601.1 |  |
|  | *r4* | Dihydrofolate synthase | 6.3.2.12 | QFY78103.1 |  |
|  | *r5* | Dihydrofolate synthase/ Folylpolyglutamate synthase | 6.3.2.12/6.3.2.17 | QFY78103.1 |  |
|  | *dedD* | DedD protein |  | QFY78102.1 |  |
|  | *dedE* | Colicin V production protein |  | QFY78101.1 |  |
|  | *purF* | Amidophosphoribosyltransferase | 2.4.2.14 | QFY78100.1 |  |
| Marinocine | *lodA* | Lysine-epsilon oxidase antimicrobial protein LodA | 1.4.3.20 | QFY78287.1 |  |
|  | *lodB* | Dehydrogenase flavoprotein LodB |  | QFY78286.1 |  |
| **Micobacterium Virulence Operons** | | | | | |
| SSU ribosomal | *rv0682* | SSU ribosomal protein S12p (S23e) |  | QFY79615.1 |  |
|  | *rv0683* | SSU ribosomal protein S7p (S5e |  | QFY79614.1 |  |
|  | *rv0684* | Translation elongation factor G |  | QFY78112.1 |  |
|  |  |  |  | QFY79613.1 |  |
|  | *rv0685* | Translation elongation factor Tu |  | QFY79630.1 |  |
|  |  |  |  | QFY79630.1 |  |
|  |  |  |  | QFY79630.1 |  |
|  |  |  |  | QFY79630.1 |  |
|  |  |  |  | QFY79612.1 |  |
| DNA transcription | *rv0667* | DNA-directed RNA polymerase beta subunit | 2.7.7.6 | QFY79623.1 |  |
|  | *rv0668* | DNA-directed RNA polymerase beta' subunit | 2.7.7.6 | QFY79622.1 |  |
| Quinolinate biosynthesis | *rv1594* | Quinolinate synthetase | 2.5.1.72 | QFY77776.1 |  |
|  | *rv1595* | L-aspartate oxidase | 1.4.3.16 | QFY77775.1 |  |
|  | *rv1596* | Quinolinate phosphoribosyltransferase [decarboxylating] | 2.4.2.19 | QFY77332.1 |  |
| LSU ribosomal | *rv1641* | Translation initiation factor 3 |  | QFY77888.1 |  |
|  | *rv1642* | LSU ribosomal protein L35p |  | QFY77900.1 |  |
|  | *rv1643* | LSU ribosomal protein L20p |  | QFY77901.1 |  |
| **Copper** | | | | | |
| Homeostasis | *crtR* | Cu(I)-responsive transcriptional regulator |  | QFY78115.1 |  |
|  | *cia* | Copper-translocating P-type ATPase | 3.6.3.4 | QFY78036.1 |  |
|  |  |  |  | QFY79818.1 |  |
|  |  |  |  | QFY77256.1 |  |
|  | *clfA* | Multidrug resistance transporter, Bcr/CflA family |  | QFY78541.1 |  |
|  |  |  |  | QFY79531.1 |  |
|  | *mo* | Multicopper oxidase |  | QFY78047.1 |  |
|  | *hl* | Cytochrome c heme lyase subunit CcmF |  | QFY76762.1 |  |
|  |  |  |  | QFY79702.1 |  |
|  | *Crd* | Copper resistance protein D |  | QFY77714.1 |  |
|  | *copG* | CopG protein |  | QFY79022.1 |  |
| Tolerance | *cutA* | Periplasmic divalent cation tolerance protein CutA |  | QFY79554.1 |  |
|  | *cutE* | Copper homeostasis protein CutE |  | QFY76812.1 |  |
|  | *corC* | Magnesium and cobalt efflux protein CorC |  | QFY76479.1 |  |
|  |  |  |  | QFY76813.1 |  |
|  |  |  |  | QFY77062.1 |  |
| **Arsenic** | | | | | |
|  | *arsB* | Arsenic efflux pump protein |  | QFY79043.1 |  |
|  | *arsC* | Arsenate reductase | 1.20.4.1 | QFY78061.1 |  |
|  |  |  |  | QFY79044.1 |  |
|  | *acr3* | Arsenical-resistance protein ACR3 |  | QFY79766.1 |  |
|  | *arsH* | Arsenic resistance protein ArsH |  | QFY79866.1 |  |
| **Cobalt-Zinc-Cadmium** | | | | | |
|  | *czcD* | Cobalt-zinc-cadmium resistance protein CzcD |  | QFY76675.1 |  |
|  | *czcA* | Cobalt-zinc-cadmium resistance protein CzcA |  | QFY79156.1 |  |
|  |  |  |  | QFY79155.1 |  |
|  | *cusB* | Probable Co/Zn/Cd efflux system membrane fusion protein |  | QFY79849.1 |  |
|  |  |  |  | QFY78804.1 |  |
|  |  |  |  | QFY76808.1 |  |
|  |  |  |  | QFY79157.1 |  |
|  |  |  |  | QFY79011.1 |  |
|  | *cusA* | Cation efflux system protein CusA |  | QFY79156.1 |  |
|  |  |  |  | QFY79155.1 |  |
|  | *czrR* | DNA-binding heavy metal response regulator |  | QFY78808.1 |  |
|  | *trcD* | Transcriptional regulator, MerR family |  | QFY79650.1 |  |
|  |  |  |  | QFY77470.1 |  |
|  |  |  |  | QFY79505.1 |  |
|  |  |  |  | QFY79326.1 |  |
|  |  |  |  | QFY77255.1 |  |
|  |  |  |  | QFY76196.1 |  |
|  |  |  |  | QFY77905.1 |  |
| **Iron** | | | | | |
| Aquisition | *pitA* | Ferric iron ABC transporter, iron-binding protein |  | QFY78006.1 |  |
|  |  |  |  | QFY78294.1 |  |
|  |  |  |  | QFY77078.1 |  |
|  | *pitD* | Ferric iron ABC transporter, ATP-binding protein |  | QFY78004.1 |  |
|  |  |  |  | QFY78291.1 |  |
|  |  |  |  | QFY78890.1 |  |
|  | *pitC* | Ferric iron ABC transporter, permease protein |  | QFY78005.1 |  |
|  |  |  |  | QFY78292.1 |  |
|  |  |  |  | QFY77079.1 |  |
|  |  |  |  | QFY78889.1 |  |
|  |  |  |  | QFY77771.1 |  |
| Heme, hemin uptake and utilization | *hutX* | Putative heme iron utilization protein |  | QFY79199.1 |  |
|  | *fhuC* | Ferrichrome transport ATP-binding protein FhuC | (TC) 3.A.1.14.3 | QFY76472.1 |  |
|  | *tonB* | Ferric siderophore transport system, periplasmic binding protein TonB |  | QFY78258.1 |  |
|  |  |  |  | QFY79885.1 |  |
|  | *eftB* | Electron transfer flavoprotein, beta subunit |  | QFY78406.1 |  |
|  | *rp2* | Outer membrane receptor proteins, mostly Fe transport |  | QFY79478.1 |  |
|  | *parA* | Paraquat-inducible protein A |  | QFY79760.1 |  |
|  | *parB* | Paraquat-inducible protein B |  | QFY77590.1 |  |
| Hemin transport | *rec* | Outer membrane receptor proteins, mostly Fe transport |  | QFY79478.1 |  |
|  | *tonB* | Ferric siderophore transport system, periplasmic binding protein TonB |  | QFY78258.1 |  |
|  |  |  |  | QFY79885.1 |  |
| **Osmotic Stress** | | | | | |
| Osmotic Stress | *osmB* | Osmotically inducible lipoprotein B precursor |  | QFY76874.1 |  |
|  | *yciM* | Heat shock (predicted periplasmic) protein YciM, precursor |  | QFY78413.1 |  |
| Osmoregulation | *aqua* | Aquaporin Z |  | QFY77413.1 |  |
|  | *ompA* | Outer membrane protein A precursor |  | QFY78423.1 |  |
|  |  |  |  | QFY78826.1 |  |
|  | *osmY* | Osmotically inducible protein OsmY |  | QFY79451.1 |  |
| Ectoine biosynthesis | *ectA* | L-2,4-diaminobutyric acid acetyltransferase | 2.3.1.- | QFY78500.1 |  |
|  | *ectB* | Diaminobutyrate-pyruvate aminotransferase | 2.6.1.46 | QFY78499.1 |  |
|  | *ectC* | L-ectoine synthase | 4.2.1.- | QFY78498.1 |  |
|  | *ectD* | Ectoine hydroxylase | 1.17.-.- | QFY78497.1 |  |
|  | *ectR* | Putative regulatory protein associated with the ectoine operon |  | QFY78501.1 |  |
| Synthesis of osmoregulated periplasmic glucans | *mdoB* | Phosphoglycerol transferase I | 2.7.8.20 | QFY77014.1 |  |
| Choline and Betaine Uptake and Betaine Biosynthesis | *betA* | Choline dehydrogenase | 1.1.99.1 | QFY78722.1 |  |
|  | *betB* | Betaine aldehyde dehydrogenase | 1.2.1.8 | QFY79524.1 |  |
|  | *betT* | High-affinity choline uptake protein BetT |  | QFY78802.1 |  |
|  | *betC* | Choline-sulfatase | 3.1.6.6 | QFY78977.1 |  |
|  | *proU* | L-proline glycine betaine ABC transport system permease protein ProV | (TC) 3.A.1.12.1 | QFY78537.1 |  |
|  |  |  |  | QFY78538.1 |  |
|  |  |  |  | QFY78539.1 |  |
|  |  |  |  | QFY79610.1 |  |
|  |  |  |  | QFY79609.1 |  |
|  | *sox* | Sarcosine oxidase beta subunit | 1.5.3.1 | QFY77948.1 |  |
|  | *gbcA* | GbcA Glycine betaine demethylase subunit A |  | QFY76875.1 |  |
| **Cold / Heat Shock Stress** | | | | | |
| Cold shock | *cspC* | Cold shock protein CspC |  | QFY79682.1 |  |
|  | *cspD* | Cold shock protein CspD |  | QFY77587.1 |  |
| Heat Shock | *yggX* | Probable Fe(2+)-trafficking protein YggX |  | QFY77217.1 |  |
|  | *RNAse* | Ribonuclease PH | 2.7.7.56 | QFY77998.1 |  |
|  | *rdgB* | Nucleoside 5-triphosphatase RdgB (dHAPTP, dITP, XTP-specific) | 3.6.1.15 | QFY77999.1 |  |
|  | *lepA* | Translation elongation factor LepA |  | QFY78580.1 |  |
|  | *SAM* | Radical SAM family enzyme, similar to coproporphyrinogen III oxidase, oxygen-independent, clustered with nucleoside-triphosphatase RdgB |  | QFY79790.1 |  |
|  | *rsmE* | Ribosomal RNA small subunit methyltransferase E | 2.1.1.- | QFY76846.1 |  |
|  | *L11+M* | Ribosomal protein L11 methyltransferase | 2.1.1.- | QFY79089.1 |  |
|  | *hrcA* | Heat-inducible transcription repressor HrcA |  | QFY78708.1 |  |
|  | *dnaJ* | Chaperone protein DnaJ |  | QFY78704.1 |  |
|  | *dnaK* | Chaperone protein DnaK |  | QFY78705.1 |  |
|  | *grpE* | Heat shock protein GrpE |  | QFY78706.1 |  |
|  | *smpB* | tmRNA-binding protein SmpB |  | QFY78075.1 |  |
|  | *hsp15* | ibosome-associated heat shock protein implicated in the recycling of the 50S subunit (S4 paralog) |  | QFY76635.1 |  |
|  | *rpoH* | RNA polymerase sigma factor RpoH |  | QFY79479.1 |  |
|  | *rsmL* | rRNA small subunit methyltransferase I |  | QFY79217.1 |  |
| **Oxidative Stress** | | | | | |
| Oxidative stress | *NO* | Nitrite-sensitive transcriptional repressor NsrR |  | QFY76777.1 |  |
|  |  |  |  | QFY77759.1 |  |
|  | *Fe* | Non-specific DNA-binding protein Dps / Iron-binding ferritin-like antioxidant protein / Ferroxidase | 1.16.3.1 | QFY79791.1 |  |
|  | *Redox* | Non-specific DNA-binding protein Dps / Iron-binding ferritin-like antioxidant protein / Ferroxidase | 1.16.3.1 | QFY79791.1 |  |
|  | *Fr* | Non-specific DNA-binding protein Dps / Iron-binding ferritin-like antioxidant protein / Ferroxidase | 1.16.3.1 | QFY79791.1 |  |
|  | *H2O2* | Hydrogen peroxide-inducible genes activator |  | QFY78010.1 |  |
|  | *SO* | Redox-sensitive transcriptional activator SoxR |  | QFY78803.1 |  |
|  | *fur_zur* | Ferric uptake regulation protein FUR |  | QFY78711.1 |  |
|  |  |  |  | QFY77689.1 |  |
|  | *fnr* | transcriptional regulator, Crp/Fnr family |  | QFY78375.1 |  |
|  |  |  |  | QFY76395.1 |  |
|  |  |  |  | QFY79148.1 |  |
|  | *sO* | Paraquat-inducible protein A and B |  | QFY79760.1 |  |
|  |  |  |  | QFY77590.1 |  |
|  | *ahpC* | Alkyl hydroperoxide reductase subunit C-like protein |  | QFY78403.1 |  |
|  |  |  |  | QFY76617.1 |  |
|  | *prxCat* | Catalase | 1.11.1.6 | QFY79664.1 |  |
|  | *zur* | Zinc uptake regulation protein ZUR |  | QFY76564.1 |  |
|  | *sodA* | Manganese superoxide dismutase | 1.15.1.1 | QFY77755.1 |  |
|  | *sodB* | Superoxide dismutase [Fe] | 1.15.1.1 | QFY77583.1 |  |
|  | *sodC* | Superoxide dismutase [Cu-Zn] precursor | 1.15.1.1 | QFY79308.1 |  |
| Protection | *sodA* | Manganese superoxide dismutase | 1.15.1.1 | QFY77755.1 |  |
|  | *sodB* | Superoxide dismutase [Fe] | 1.15.1.1 | QFY77583.1 |  |
|  | *sodC* | Superoxide dismutase [Cu-Zn] precursor | 1.15.1.1 | QFY79308.1 |  |
|  | *hpII* | Catalase | 1.11.1.6 | QFY79664.1 |  |
|  | *ccp* | Cytochrome c551 peroxidase | 1.11.1.5 | QFY76775.1 |  |
|  |  |  |  | QFY77403.1 |  |
| Glutathione: non-redox reactions | *gt1* | Glutathione S-transferase | 2.5.1.18 | QFY78022.1 |  |
|  |  |  |  | QFY78062.1 |  |
|  |  |  |  | QFY78302.1 |  |
|  |  |  |  | QFY78452.1 |  |
|  |  |  |  | QFY78526.1 |  |
|  |  |  |  | QFY77089.1 |  |
|  |  |  |  | QFY79508.1 |  |
|  |  |  |  | QFY77712.1 |  |
|  |  |  |  | QFY76230.1 |  |
|  | *gt2* | Glutathione S-transferase family protein |  | QFY78483.1 |  |
|  |  |  |  | QFY77717.1 |  |
|  | *gloA* | Lactoylglutathione lyase | 4.4.1.5 | QFY76838.1 |  |
|  |  |  |  | QFY76925.1 |  |
|  |  |  |  | QFY79663.1 |  |
|  | *gloB* | Hydroxyacylglutathione hydrolase | 3.1.2.6 | QFY79014.1 |  |
|  | *sam* | SAM-dependent methyltransferase | 2.1.1.- | QFY79013.1 |  |
| **Periplasmic Stress** | | | | | |
|  | *degS* | Outer membrane stress sensor protease DegS |  | QFY79405.1 |  |
|  | *rseA* | Sigma factor RpoE negative regulatory protein RseA |  | QFY78583.1 |  |
|  | *skp* | Outer membrane protein H precursor |  | QFY78086.1 |  |
|  | *surA* | Survival protein SurA precursor (Peptidyl-prolyl cis-trans isomerase SurA) | 5.2.1.8 | QFY76922.1 |  |
|  |  |  |  | QFY79291.1 |  |
|  | *depQ* | HtrA protease/chaperone protein |  | QFY78581.1 |  |
|  | *rseB* | Sigma factor RpoE negative regulatory protein RseB precursor |  | QFY78582.1 |  |
| **Stress Response** | | | | | |
| Flavohaemoglobin | *hmpX* | Flavohemoprotein (Hemoglobin-like protein) (Flavohemoglobin) (Nitric oxide dioxygenase) | 1.14.12.17 | QFY79510.1 |  |
|  |  |  |  | QFY77700.1 |  |
|  | *NOR* | Nitric-oxide reductase, quinol-dependent | 1.7.99.7 | QFY79146.1 |  |
|  | *ppdB* | 3-phenylpropionate dioxygenase, beta subunit | 1.14.12.19 | QFY78756.1 |  |
|  | *ppdA* | 3-phenylpropionate dioxygenase, alpha subunit | 1.14.12.19 | QFY78755.1 |  |
| Bacterial hemoglobin | *GGDEF* | diguanylate cyclase/phosphodiesterase (GGDEF & EAL domains) with PAS/PAC sensor(s) |  | QFY78318.1 |  |
|  |  |  |  | QFY78773.1 |  |
|  |  |  |  | QFY76441.1 |  |
|  |  |  |  | QFY76806.1 |  |
|  |  |  |  | QFY76811.1 |  |
|  |  |  |  | QFY76382.1 |  |
|  |  |  |  | QFY77216.1 |  |
|  |  |  |  | QFY79905.1 |  |
|  |  |  |  | QFY78828.1 |  |
|  |  |  |  | QFY77557.1 |  |
| Hfl operon | *hfq* | RNA-binding protein Hfq |  | QFY78328.1 |  |
|  | *hflX* | GTP-binding protein HflX |  | QFY78327.1 |  |
|  | *hflK* | HflK protein |  | QFY78326.1 |  |
|  | *hflC* | HflC protein |  | QFY78325.1 |  |
| Carbon Starvation | *cstA* | Carbon starvation protein A |  | QFY78491.1 |  |
|  | *sspA* | Stringent starvation protein A |  | QFY79410.1 |  |
|  | *sspB* | ClpXP protease specificity-enhancing factor / Stringent starvation protein B |  | QFY79411.1 |  |
| **DNA Repair** | | | | | |
| Bacterial DNA repair | *recA* | RecA protein |  | QFY78230.1 |  |
|  | *radA* | DNA repair protein RadA |  | QFY77206.1 |  |
|  | *radC* | DNA repair protein RadC |  | QFY78528.1 |  |
|  | *rdgC* | DNA recombination-dependent growth factor C |  | QFY78234.1 |  |
|  |  |  |  | QFY77346.1 |  |
|  |  |  |  | QFY77346.1 |  |
|  |  |  |  | QFY77784.1 |  |
|  | *xseA* | Exodeoxyribonuclease VII large subunit | 3.1.11.6 | QFY77582.1 |  |
|  | *xseB* | Exodeoxyribonuclease VII small subunit | 3.1.11.6 | QFY77183.1 |  |
|  | *lexA* | SOS-response repressor and protease LexA | 3.4.21.88 | QFY77638.1 |  |
|  | *dinP* | DNA polymerase IV | 2.7.7.7 | QFY76273.1 |  |
|  | *imuB* | DNA polymerase IV-like protein ImuB |  | QFY77962.1 |  |
|  | *ssb* | Single-stranded DNA-binding protein |  | QFY77348.1 |  |
|  |  |  |  | QFY79539.1 |  |
|  |  |  |  | QFY77494.1 |  |
|  |  |  |  | QFY77786.1 |  |
|  | *exoIII* | Exodeoxyribonuclease III | 3.1.11.2 | QFY78132.1 |  |
|  |  |  |  | QFY79143.1 |  |
|  | *ogt* | Methylated-DNA-protein-cysteine methyltransferase | 2.1.1.63 | QFY76512.1 |  |
|  |  |  |  | QFY76534.1 |  |
|  |  |  |  | QFY76767.1 |  |
|  | *ada* | ADA regulatory protein |  | QFY76534.1 |  |
|  |  |  |  | QFY76767.1 |  |
|  | *alkB* | Alkylated DNA repair protein AlkB |  | QFY78304.1 |  |
|  | *recN* | DNA repair protein RecN |  | QFY78710.1 |  |
|  | *rmuC* | DNA recombination protein RmuC |  | QFY76379.1 |  |
|  | *mutY* | A/G-specific adenine glycosylase | 3.2.2.- | QFY79873.1 |  |
| Uracyl-DNA glycosylase | *udg1* | Uracil-DNA glycosylase, family 1 |  | QFY76427.1 |  |
|  | *udg4* | Uracil-DNA glycosylase, family 4 |  | QFY78624.1 |  |
|  | *udg6* | Uracil-DNA glycosylase, family 6 |  | QFY76939.1 |  |
|  | *fused* | Domain often clustered or fused with uracil-DNA glycosylase / Uracil-DNA glycosylase, putative family 6 |  | QFY76939.1 |  |
| *recA/MultiL-S* | *mutS* | DNA mismatch repair protein MutS |  | QFY77036.1 |  |
|  | *mutL* | DNA mismatch repair protein MutL |  | QFY76946.1 |  |
|  | *recA* | RecA protein |  | QFY78230.1 |  |
|  | *Rep* | C-terminal domain of CinA type S; Protein Implicated in DNA repair function with RecA and MutS |  | QFY78942.1 |  |
|  | *recX* | Regulatory protein RecX |  | QFY78229.1 |  |
| *recFOR* pathway | *recO* | DNA recombination and repair protein RecO |  | QFY78576.1 |  |
|  | *recR* | Recombination protein RecR |  | QFY76979.1 |  |
|  | *recQ* | ATP-dependent DNA helicase RecQ |  | QFY76884.1 |  |
|  | *recJ* | Single-stranded-DNA-specific exonuclease RecJ |  | QFY77657.1 |  |
|  | *ssb* | Single-stranded DNA-binding protein |  | QFY77348.1 |  |
|  |  |  |  | QFY79539.1 |  |
|  |  |  |  | QFY77494.1 |  |
|  |  |  |  | QFY77786.1 |  |
|  | *recA* | RecA protein |  | QFY78230.1 |  |
| *uvrABC* system | *uvrA* | Excinuclease ABC subunit A |  | QFY79541.1 |  |
|  | *uvrA-d* | Excinuclease ABC subunit A, dimeric form |  | QFY76869.1 |  |
|  | *uvrB* | Excinuclease ABC subunit B |  | QFY77641.1 |  |
|  | *uvrC* | Excinuclease ABC subunit C |  | QFY78387.1 |  |
| Photolyase | *phrB* | Deoxyribodipyrimidine photolyase | 4.1.99.3 | QFY78875.1 |  |
