# Supplementary material for: Complete genome sequence and analysis of Alcaligenes faecalis strain Mc250, a new potential plant bioinoculant
Source: PLoS One. 2020 Nov 5;15(11):e0241546. doi: 10.1371/journal.pone.0241546 (PMC7643998; doi:10.1371/journal.pone.0241546)
Supplement: S4 Table — (DOCX) [file pone.0241546.s008.docx]

**S4 Table** Identified secondary metabolite clusters in *AfMc250* genome

| **Cluster** | **Type** | **From** | **To** | **Most similar known cluster** | **% genes similarity** | **MIBiG BGC-ID** | **Fig.Ref.** |
| --- | --- | --- | --- | --- | --- | --- | --- |
| [1](https://antismash.secondarymetabolites.org/upload/bacteria-98f0b2fd-cf33-4822-bdc4-1f24c1a5f6be/index.html#cluster-6) | [Resorcinol](http://antismash.secondarymetabolites.org/help#resorcinol)-[Nrps](http://antismash.secondarymetabolites.org/help#nrps) | 308897 | 385213 | Bacillibactin biosynthetic gene cluster | 23 | [BGC0000309_c1](http://mibig.secondarymetabolites.org/repository/BGC0000309/index.html) | 4A |
| [2](https://antismash.secondarymetabolites.org/upload/bacteria-98f0b2fd-cf33-4822-bdc4-1f24c1a5f6be/index.html#cluster-10) | [Terpene](http://antismash.secondarymetabolites.org/help#terpene) | 1224612 | 1246342 | Burkholderic acid biosynthetic gene cluster | 13 | [BGC0001120_c1](http://mibig.secondarymetabolites.org/repository/BGC0001120/index.html) | 4D |
| [3](https://antismash.secondarymetabolites.org/upload/bacteria-98f0b2fd-cf33-4822-bdc4-1f24c1a5f6be/index.html#cluster-2) | [Ectoine](http://antismash.secondarymetabolites.org/help#ectoine) | 2723292 | 2733684 | Ectoine biosynthetic gene cluster | 75 | [BGC0000853_c1](http://mibig.secondarymetabolites.org/repository/BGC0000853/index.html) | 4C |
| [4](https://antismash.secondarymetabolites.org/upload/bacteria-98f0b2fd-cf33-4822-bdc4-1f24c1a5f6be/index.html#cluster-15) | [T1pks](http://antismash.secondarymetabolites.org/help#t1pks)-[Cf_saccharide](http://antismash.secondarymetabolites.org/help#cf_saccharide) | 3768090 | 3815664 | Emulsan biosynthetic gene cluster | 9 | [BGC0000760_c1](http://mibig.secondarymetabolites.org/repository/BGC0000760/index.html) | 4B |
